# Genomic characterization of three novel Basilisk-like phages infecting *Bacillus anthracis*

**DOI:** 10.1186/s12864-018-5056-4

**Published:** 2018-09-18

**Authors:** J. Farlow, D. Bolkvadze, L. Leshkasheli, I. Kusradze, A. Kotorashvili, N. Kotaria, N. Balarjishvili, L. Kvachadze, M. Nikolich, M. Kutateladze

**Affiliations:** 1 George Eliava Institute for Bacteriophages, Microbiology and Virology, Tbilisi, Georgia; 2 Lugar Center for Public Health Research at National Center for Disease Contro, Tbilisi, Georgia; 3 Farlow Scientific Consulting Company, LLC, Lewiston, UT USA; 4 Department of Bacteriophage Therapeutics, Bacterial Diseases Branch, Walter Reed Army Institute of Research, Silver Spring, Silver Spring, MD USA

**Keywords:** Phage genome, Phage evolution, *Bacillus anthracis*

## Abstract

**Background:**

In the present study, we sequenced the complete genomes of three novel bacteriophages v_B-Bak1, v_B-Bak6, v_B-Bak10 previously isolated from historical anthrax burial sites in the South Caucasus country of Georgia. We report here major trends in the molecular evolution of these phages, which we designate as “Basilisk-Like-Phages” (BLPs), and illustrate patterns in their evolution, genomic plasticity and core genome architecture.

**Results:**

Comparative whole genome sequence analysis revealed a close evolutionary relationship between our phages and two unclassified *Bacillus cereus* group phages, phage Basilisk, a broad host range phage (Grose JH et al., J Vir. 2014;88(20):11846-11860) and phage PBC4, a highly host-restricted phage and close relative of Basilisk (Na H. et al. FEMS Microbiol. letters. 2016;363(12)). Genome comparisons of phages v_B-Bak1, v_B-Bak6, and v_B-Bak10 revealed significant similarity in sequence, gene content, and synteny with both Basilisk and PBC4. Transmission electron microscopy (TEM) confirmed the three phages belong to the *Siphoviridae* family. In contrast to the broad host range of phage Basilisk and the single-strain specificity of PBC4, our three phages displayed host specificity for *Bacillus anthracis*. *Bacillus* species including *Bacillus cereus, Bacillus subtilis, Bacillus anthracoides,* and *Bacillus megaterium* were refractory to infection.

**Conclusions:**

Data reported here provide further insight into the shared genomic architecture, host range specificity, and molecular evolution of these rare *B. cereus* group phages. To date, the three phages represent the only known close relatives of the Basilisk and PBC4 phages and their shared genetic attributes and unique host specificity for *B. anthracis* provides additional insight into candidate host range determinants.

**Electronic supplementary material:**

The online version of this article (10.1186/s12864-018-5056-4) contains supplementary material, which is available to authorized users.

## Background

Members of the *Bacillus* genus are ubiquitous, spore forming Gram-positive bacilli that include the food-borne pathogen *Bacillus cereus* and the causative agent of anthrax, *Bacillus anthracis* [1]. The taxonomy of the *Bacillus cereus* group (*sensu* *lato*) currently designates seven closely related species: *B. cereus* (*sensu stricto*)*, B. mycoides, B. pseudomycoides, B. thuringiensis, B. weihenstephanensis, B. toyonensis,* and *B. anthracis* [2]. The *B. cereus* group is genetically homogeneous [1]. Bacteriophages with narrow host specificity within the *B. cereus* group have shown utility for typing and possess potential for drug discovery and biocontrol [3]. Phages are also considered important mediators of microbial genetic exchange with potential to impact both the adaptation and virulence of their respective hosts in addition to influencing host ecology and evolution [4, 5].

*Bacillus* phages are less well-characterized than phages infecting *Mycobacterium* and members of the Gram-negative *Enterobacteriaceae* family [6]*.* Phages infecting *B. cereus* group members display significant diversity in morphology and host specificity, representing multiple taxonomic families including the *Myoviridae*, *Siphoviridae, Podoviridae*, and *Tectiviridae* [7]. While typing phages for *B. anthracis* and *B. subtilis* have been well studied, the wide variety of additional bacteriophages infecting the *Bacillus* group remain largely uncharacterized [6]. The rapidly expanding field of phage genomics, driven by low-cost high-throughput whole genome sequencing, has provided unprecedented opportunities to characterize bacteriophage population structure. In addition, genome-level surveys facilitate better assessments of the potential for modular evolution of phages, including their impact on the distribution of microbial virulence factors and identification of candidate host range determinants [6].

Recently, whole genome nucleotide and proteome comparisons of 93 *Bacillus* phages revealed major patterns in the population structure of the *Bacillus* phages, resolving 12 highly diverse evolutionary groups (Clusters) encompassing 28 subclusters and numerous singletons [6]. Such data illustrate trends in population structure and provide a foundation for assessing the extent by which host relatedness influences phage evolution. Additional phage discovery efforts will further refine inferred population structures and facilitate accurate phylogenetic placement of novel singleton phages.

In the present study, we sequenced the complete genomes of three novel bacteriophages v_B-Bak1, v_B-Bak6, v_B-Bak10 previously isolated from historical anthrax burial sites in the South Caucasus country of Georgia. Comparative whole genome sequence analysis revealed a close evolutionary relationship between the three newly characterized phages and phage Basilisk, a broad host range *B. cereus* group phage [8] as well as phage PBC4, a highly host-restricted *B. cereus* phage and close relative of Basilisk [9]. We report here major trends in the molecular evolution of these phages, which we designate as “Basilisk-Like-Phages” (BLPs), and illustrate patterns in their evolution, genomic plasticity and core genome architecture.

## Methods

### Host range testing

The vaccine strain of *B. anthracis* 34F_2_ was used for phage propagation. Phages in high concentration were prepared using the Double-Layer Agar (DLA) technique [10]. For stability testing, phages underwent titration by Appelmans method in liquid medium [11]. The Appelmans serial passage method was used to expand the host range of bacteriophage mixtures in liquid medium [10, 11]. In vitro screening of bacterial strains for susceptibility to phages was performed using the “spot test” method [12]. A bacterial lawn of fresh bacterial cultures with a concentration of 10^8^ colony forming units (cfu)/ml was prepared on Trypticase Soy Agar plates. 10 μL of bacteriophage suspensions with a titer of 10^7^ plaques forming units (pfu)/ml were placed on the bacterial lawn and incubated overnight (18–24 h) at 37 °C [12].

Morphology of bacteriophage virions were examined by Transmission Electron Microscopy (TEM) (Jeol 100 SX). EMS300-CU slides were used for samples. Negative contrasting of preparations were performed with uranyl acetate.

### Genome sequencing and phylogenetics

Genomic DNA was extracted from purified phage preparations using a Qiagen DNA mini prep kit and sheared to 350 bp using the Covaris M220 L. Whole-genome shotgun sequencing on the Illumina MiSeq platform was performed. Mapped and de novo read assemblies were analyzed using CLC Bio (http: //www.clcbio.com) and Geneious version 7.0 [13] using the Basilisk (KC595511) and PBC4 (JQ_19704.1) genomes as references. De novo assembly yielded single whole-genome-level contiguous sequences for each of the three phage genomes. Genome sizes of the three phages were approximately 80,764 bp (v_B-Bak1), 80,764 bp (v_B-Bak6), and 82,931 bp (v_B-Bak10) (Table 1). The RAST annotation server and the NCBI Prokaryotic Genome Annotation Pipeline were used for functional annotation using manual annotation. The Basilisk (KC595511.2) and PBC4 (KT070866) genomes were used as references. Geneious version 7.0 [13] was used to prepare all illustrations of protein and nucleic acid alignments. PyMOl 2.0.4 was used to represent the putative 3D structure of the CP-domain protein. The genomic sequences of each phage were determined and deposited in GenBank with the following Genbank nucleotide sequence accession numbers: v_B-Bak1 (MG967616), v_B-Bak6 (MG967617), and v_B-Bak10 (MG967618). Unrooted Neighbor-Joining phylogenies were performed based per best model fit analysis (JC and LG + G + I models) determined from Bayesian Information Criterion (BIC) in MEGA 5 [14]. Branch lengths were measured in number of substitutions per site and bootstrap (BS) performed with 1000 replicates.

**Table 1:** Genome attributes of the three phages and reference genomes of phages Basilisk and PBC4

Phage	Host	Size	%GC	No. ORFs	tRNAs	Accession No.	Location	Reference
v_B-Bak1	*B. anthracis*	80,764	33.9	139	2	MG967616	Kutaisis, country of Georgia	this paper
v_B-Bak6	*B. anthracis*	80,764	33.9	139	2	MG967617	Kutaisis, country of Georgia	this paper
v_B-Bak10	*B. anthracis*	82,931	33.8	142	3	MG967618	Kutaisis, country of Georgia	this paper
Basilisk	*B. cereus*	81,790	33.9	140	2	KC59551	Kutaisis, country of Georgia Utah, USA	Grose et al. 2014
PBC4	*B. cereus*	80,647	34	123	2	KT070866	South Korea	Na et al. 2016

## Results

### Phage isolation and host range

Phages v_B-Bak1, v_B-Bak6, and v_B-Bak10 were isolated in a previous un-published study from soil samples recovered from cattle anthrax burial sites in western Georgia, near Maghlaki village, Kutaisi (Additional file 1: Figure S1). The isolation protocol has been described previously (Mark Adams, Bacteriophages, 1956, Interscience publishers, INC., New York). All phages and bacterial host strains were obtained from the George Eliava Institute for Bacteriophages. Each of the three phages were isolated from independent enrichment cultures containing *B. anthracis* strains STI-1, 55-VNIIVViM, 34-F2, and 34-Ikhtiman from the George Eliava Institute for Bacteriophages, Microbiology and Virology. In a separate un-published effort, the three phages had been tested for activity on a diverse panel of virulent wild-type Georgian *B. anthracis* strains from the repository of the R. Lugar Center for Public Health Research and displayed strong lytic activity.

Phage lytic ability was examined against five *Bacillus* species including *B. cereus*, *B. anthracis*, *B. subtilis, B. anthracoides,* and *B. megaterium*. Phages v_B-Bak1, v_B-Bak6, and v_B-Bak10 displayed strong lytic activity on *B. anthracis* (Table 2). *Bacillus* species including *B. cereus, B. subtilis, B. anthracoides,* and *B. megaterium* were refractory to infection. By comparison, the Basilisk phage displayed broad host range infecting *B. cereus, B. anthracis,* and *B. thuringiensis* [8] (Table 2). Phage PBC4, in contrast, exhibits pronounced host specificity for *B. cereus* strain 14,579 [9].

**Table 2:** Host specificity of phages v_B-Bak1, v_B-Bak6 and v_B-Bak10 and reference phages Basilisk and PBC4 on Bacillus hosts

Host						
Phage	*B. cereus*	*B. anthracis*	*B. thuringiensis*	*B. subtilis*	*B. mycoides*	*B. megaterium*
v_B-Bak1	–	+	–	–	–	–
v_B-Bak6	–	+	–	–	–	–
v_B-Bak10	–	+	–	–	–	–
Basilisk	+	+	+	–	–	–
PBC4	+	+	–	–	–	–

### Phage morphology

**Fig. 1:**
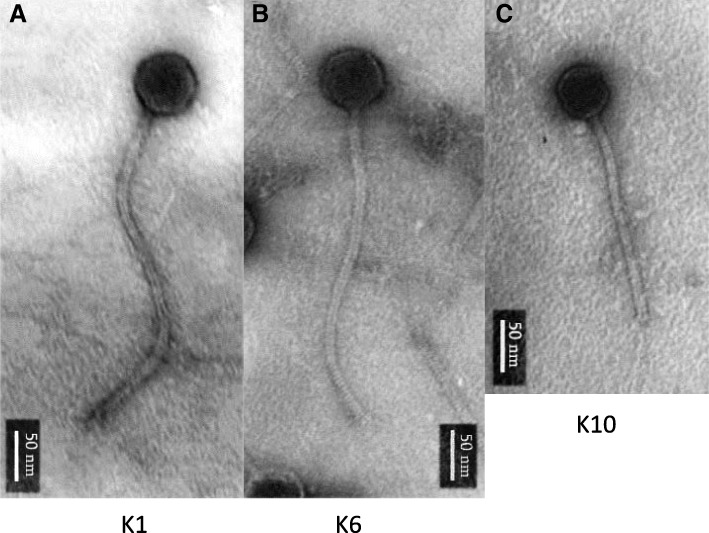
Transmission electron microscopy of the three phages. TEM images of phages v_B-Bak1 (**a**), v_B-Bak6 (**b**), and v_B-Bak10 (**c**) illustrate their morphology consistent with members of the *Siphoviridae* family

**Fig. 2:**
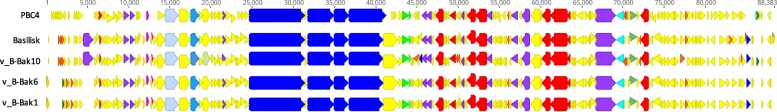
Whole genome open reading frame alignments of phage v_B-Bak1, v_B-Bak6, v_B-Bak10 and the Basilisk and PBC4 reference genomes. Color designations are according to putative function: teal, packaging; blue, structural genes; green, host lysis; red, DNA manipulation; pink tRNA genes, purple, additional functions; and yellow, hypothetical genes

Transmission electron microscopy (TEM) confirmed that the three phages possess icosahedral heads and long tails, confirming their classification as members of the *Siphoviridae* family (Fig. 1). Phages v_B-Bak1 and v_B-Bak6 were found to possess larger heads and tails (~ 64 nm/~ 357 nm) in comparison to v_B-Bak10 (55 nm/200 nm). The morphological dimensions of Basilisk and PBC4 were previously reported as ~ 72 nm and 420 nm (Basilisk) and 65 nm and 430 nm (PBC4) for corresponding heads and tails, respectively. The tape measure protein encoded by PBC4 (2251 amino acid residues) is consistent with its longer length (430 nm) compared to phage Basilisk (420 nm, 2186 residues, respectively). The TMP gene sequence length of v_B-Bak1, v_B-Bak6, and v_B-Bak10 were identical to Basilisk gp47 however the tail lengths of both v_B-Bak1 and v_B-Bak6 (357 nm, panels A and B) appeared longer than v_B-Bak10 (200 nm, panel C) (Fig. 2) and shorter than Basilisk (420 nm).

### Genome comparisons

**Fig. 3:**
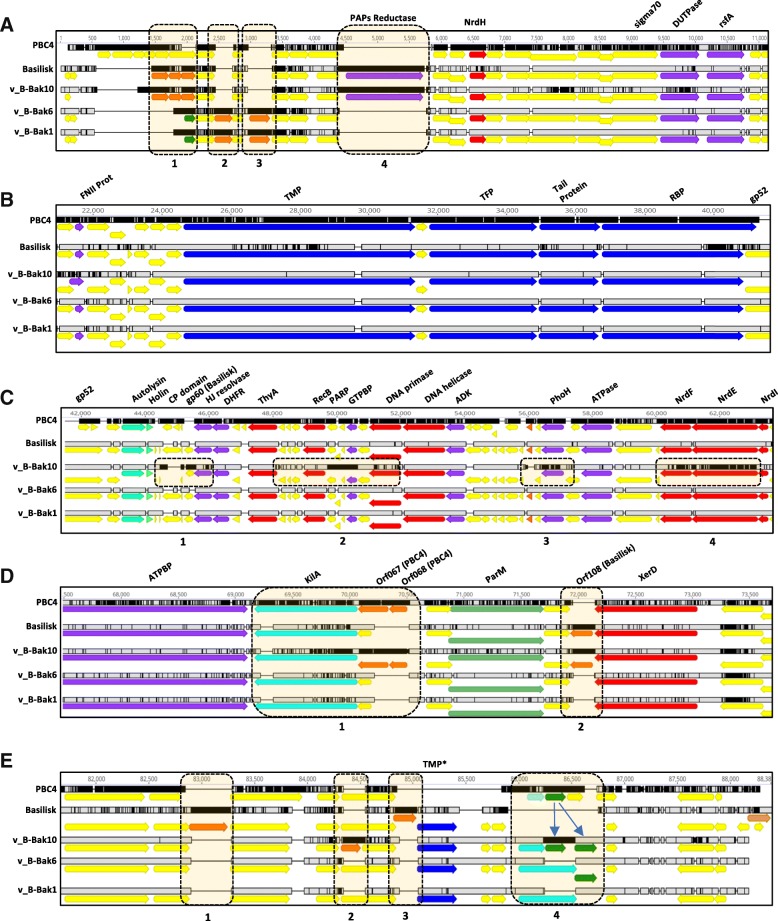
Major genomic diversity regions in the genomes of the Basilisk-Like-Phages. **a**) left terminal region InDels, **b**) structural module, **c**) nucleic acid metabolism genes, **d**) putative virulence genes, and **e**) right terminal region. Boxed Diversity Regions (DRs) in dotted orange highlight illustrate major variations among phages v_B-BaK1, v_B-BaK6, v_B-BaK10, and Basilisk

**Fig. 4:**
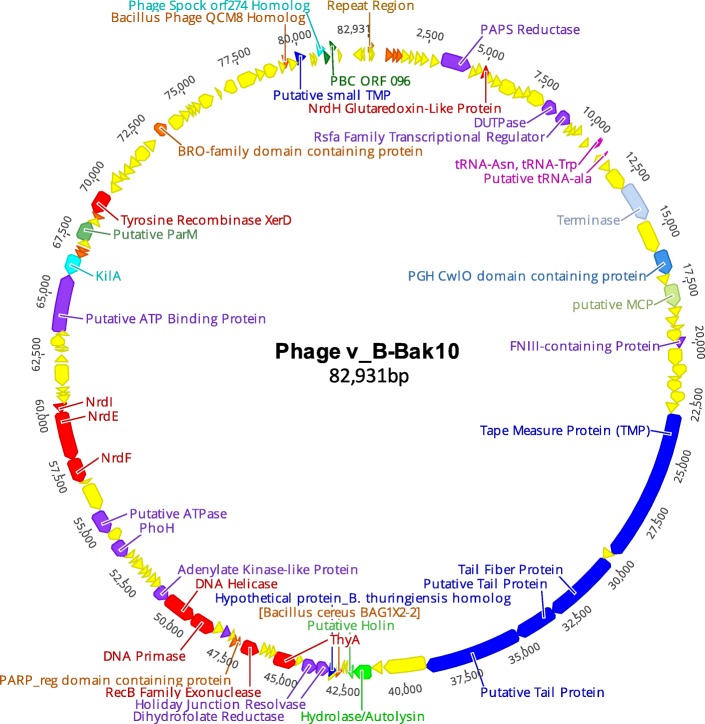
Whole genome map of phage v_B-BaK10. A circular representation of the linear phage genome was used here for illustration purposes

Phages v_B-Bak1, v_B-Bak6 and v_B-Bak10 possess dsDNA genomes of approximately ~ 80 kb (Table 1), similar to both Basilisk and PBC4 [6, 8, 9]. With the exception of minor instances of gene gain/loss the three genomes are highly syntenic with the genomes of phage Basilisk and PBC4 (Fig. 2, Additional file 2: Figure S2). Consistent with their high similarity to phage Basilisk (99%) (Additional file 3: Figure S3), the three phage genomes display a modular organization of structural and functional genes including DNA packaging, head and tail structure/morphogenesis, host lysis, and DNA replication modules common to siphophages (Figs. 3 and 4). The genomes of phages v_B-Bak1 and v_B-Bak6 are nearly identical with distinguishing single nucleotide polymorphisms (SNPs) present in only two genes, the homologs of Basilisk gp36 (putative capsid) and a hypervariable locus in gp52 (hypothetical protein). Phage v_B-Bak10 displays multiple gene insertions/deletions (indels) uniquely shared with either PBC4 or Basilisk as well as entirely unique open reading frames (ORFs) (Figs. 3a, d, and e, 4). Among the BLPs, v_B-Bak10 displays the largest genome size (82,932 bp) (Table 1). The average GC content of the v_B-Bak1, v_B-Bak6 and v_B-Bak10 phages (33%) and *B. cereus* group chromosomes (~ 35%) is consistent with the slightly lower GC content often observed in phages compared to their host chromosomes [16].

### Gene content and diversity

Gene content and diversity among the BLPs are illustrated in Fig. 3 and Additional file 1: Figure S1. The majority of sequence variations among v_B-Bak1, v_B-Bak6, v_B-Bak10 and Basilisk are localized to the left and right terminal regions of the genome corresponding to consensus residues 1-23 K (left terminal region) and 61–88.3 K (right terminal region) (Additional file 2: Figure S2). Phages v_B-Bak1, v_B-Bak6 and v_B-Bak10 possess a similar number of ORFs as Basilisk (v_B-Bak1/v_B-Bak6 = 139, v_B-Bak10 = 139, Basilisk = 140) while PBC4 contains the fewest predicted genes (*N* = 123). To illustrate trends in localized genomic diversity between the three phages and Basilisk, we arbitrarily designated short genomic regions that displays a dense number of SNPs and/or indels as diversity regions (DRs), in boxed orange highlight (Fig. 3a-e).

The three newly characterized phages (and PBC4) lacked Basilisk hypothetical proteins gp2, gp129, gp133, gp140. BLASTP searches revealed no putative homologs of Basilisk gp2 or gp140 currently in Genbank. Basilisk gp129 shows 70% similarity to a hypothetical protein encoded by select phages including *B. cereus* phage PBC2 (70% amino acid similarity) and the *B. megaterium* phage Eldridge (61% amino acid similarity). Basilisk gp133 displayed 82% amino acid similarity (85% coverage) to a hypothetical protein encoded by *B. cereus* (WP_088363480.1). We speculate genes within this set may be required to infect *B. cereus.* Phages v_B-Bak1 and v_B-Bak6 uniquely encoded three hypothetical proteins (orf3, orf5, and orf7) (Fig. 3, A-DR1–3). Orf3 encoded by v_B-Bak1 and v_B-Bak6 displays similarity to hypothetical proteins encoded by *Desulfotomaculum guttoideum* (63%) (SEU10093.1) and *Clostridium sp.* ASBs410 (59%) (EXG87698.1) while orf5 (Fig. 3, A-DR2) displays similarity to hypothetical proteins encoded by *Bacillus* phage BigBertha (54%), B4 (52%), Juglone (52%), and Spock (49%). BLASTP homology searches of v_B-Bak1 and v_B-Bak6 orf7 revealed a striking 100% similarity to *B. cereus* hypothetical protein WP_073526592.1 and 83% similarity to a multi-species hypothetical protein encoded by members of the *B. cereus* group. Directly downstream and adjacent to the KilA domain protein (Fig. 3d-DR1, yellow) phages Basilisk, v_B-Bak1, and v_B-Bak6 encode homologs to Basilisk gp104 that displays high similarity to a select group of *Bacillus* myoviruses including Deep Blue, BM15, BCP8–2, and JBP901. Phages v_B-Bak1 and v_B-Bak6 were also distinguished from each other by non-synonymous polymorphisms in Basilisk gp36 and gp52 (hypothetical proteins). It is possible variations in either or both of these genes may be responsible for the greater selectivity v_B-Bak1 displays among the *B. anthracis* hosts tested.

Phage v_B-Bak10 encoded multiple genes uniquely shared with either Basilisk or PBC4 as well as five genes not present in other phages studied here. In the left terminal region, phages v_B-Bak10 and Basilisk share three hypothetical proteins (Basilisk gp3–5, Fig. 3, A-DR1) as well as the PAPs reductase gene (Basilisk gp11, Fig. 3 A-DR4) that are absent in v_B-Bak1, v_B-Bak6 and PBC4. Within DR1, phage v_B-Bak10 (orf003) and Basilisk gp3 share 80% homology and together share 53% homology to a hypothetical protein encoded by *B. chagannorensis* (WP_035212193.1) while Basilisk gp4 is unique to Basilisk, v_B-Bak10 and PBC4 (orf104) with no homologs detected in Genbank. The closest homolog of Basilisk gp5 (hypothetical protein) and the v_B-Bak10 homolog was encoded by *Sporosarcina globispora* (44%) (WP_053433955). Phage v_B-Bak10 was also distinguished from v_B-Bak1 and v_B-Bak6 by the absence of Basilisk gp70, gp83, and gp118. Overall the genome of phage v_B-Bak10 exhibits seven unique whole gene-indels compared to phages v_B-Bak1 and v_B-Bak6. In addition, phages v_B-Bak10 and PBC4 shared genes not present in the other BLPs. Adjacent to the KilA protein (Basilisk gp103), phages v_B-Bak10 and PBC4 uniquely encoded two hypothetical proteins (PBC4 orf67 and orf68) (Fig. 3, D-DR1, orange). Phage v_B-Bak10 also encoded a homolog of PBC4 orf96 (hypothetical protein) in the right terminus of the genome (Fig. 3e-DR4, green). Genes unique to v_B-Bak10 include a putative tRNA-ala, a homolog of a CP-domain-containing protein encoded by *B. cereus* BAG1X2–2, a *B. thuringiensis* hypothetical protein homolog, a PARP regulatory domain-containing protein and a homolog of a hypothetical protein encoded by *Bacillus* phage QCM8 (Fig. 4). Lastly, v_B-Bak10 was further distinguished by the absence of an additional six genes encoding hypothetical proteins, including Basilisk gp29, gp59, gp70, gp83, gp103, and gp118.

### Phylogenetic relationships

**Fig. 5:**
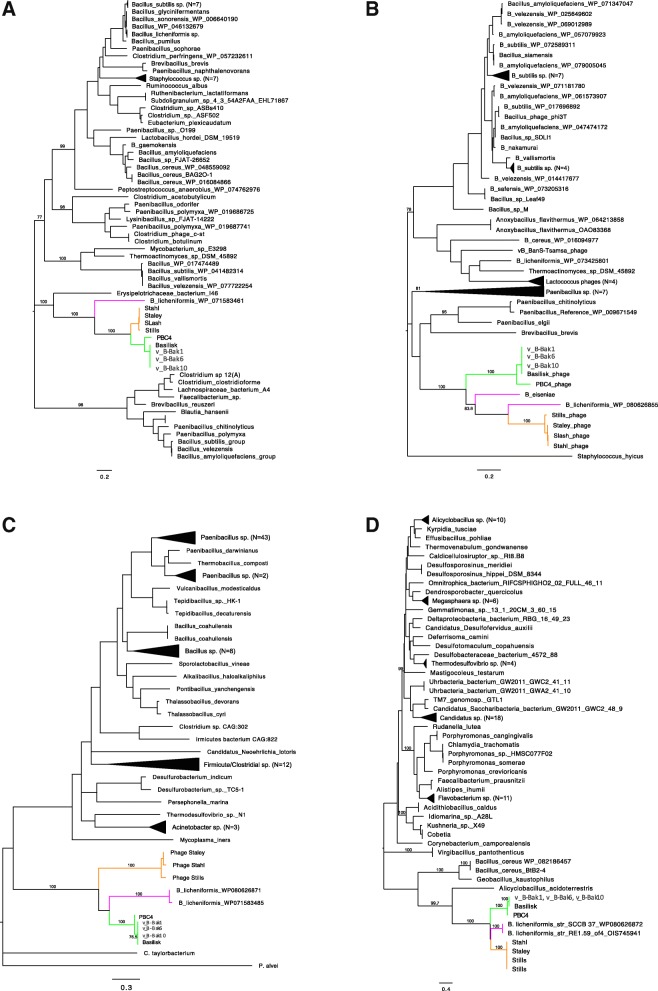
Phylogenetic relationships among Basilisk-like phages. Unrooted maximum-likelihood dendrogram derived from amino acid sequences of the phage Terminase (**a**), Tape measure protein (**b**), DNA primase (**c)**, and DNA helicase (**d**) proteins

While previous studies have used single-gene or protein phylogenies to discriminate major evolutionary assemblages [17], this approach can be can be problematic due to the mosaic nature of bacteriophage genomes. Phages v_B-Bak1, v_B-Bak6, v_B-Bak10, Basilisk and PBC4 exhibited robust phylogenetic affiliation based on the amino acid sequences of their terminases and tape measure proteins (TMPs) (Fig. 5a and b). Similar phylogenetic associations were also inferred based on replication-associated proteins (DNA primase and helicase) (Fig. 5 c and d). In each dendogram topology, phage PBC4 appears basal to other BLPs consistent with its lower overall genome-wide similarity. The BLPs also exhibit significant phylogenetic association with *B. megaterium* phages Staley, Slash, Stills and Stahl (Fig. 5a-d), consistent with previous whole genome and proteome comparison that demonstrated near neighbor status between phages Basilisk, Staley and Slash (Grose 2014b). The genomes of Stills and Stahl were not yet reported at the time of the study by Grose et al. In addition, we observed that all three of the BLPs and the four *B. megaterium* phages formed consistent evolutionary assemblages with prophage sequences from *B. licheniformis* and to a lesser extent *B. eiseniae* strains (Fig. 5). Overall, these data support a close genetic affiliation between the BLPs, *B. licheniformis* prophages and phages Stills, Stahl, Staley and Slash.

### Virion module

Genes encoding the 15 virion structural and assembly proteins identified in phage Basilisk [8] were present and syntenic in the v_B-Bak1, v_B-Bak6, v_B-Bak10, and PBC4. In all cases, DNA sequences of v_B-Bak1, v_B-Bak6, and v_B-Bak10 display higher overall sequence similarity to phage Basilisk. The genomic position and alignment of the highly conserved tape measure proteins (TMPs), tail fiber proteins (TFPs) and tail proteins is illustrated in Fig. 3b).

### Tape measure protein (TMP)

Phage TMPs facilitate phage tail length determination and promote translocation of the phage genome to the host cell cytoplasm [18, 19]. In addition, the varied domain architectures of phage TMPs are largely uncharacterized and reflect their multifunctional roles in phage biology. Consistent with other dsDNA bacteriophage genomes, the TMP genes of the BLPs are the largest gene in their corresponding genomes (PBC4 = 2250aa, others = 2185aa). The putative TMP homologs encoded by v_B-Bak1, v_B-Bak6 and v_B-Bak10 share 99% amino acid similarity to Basilisk gp47 and 92% homology to the translated product of PBC4 orf17. BLASTP homology to the full length TMP sequences of v_B-Bak1, v_B-Bak6, and v_B-Bak10 was shared only by the phage Basilisk and PBC4 TMPs while the N and C terminal regions independently display limited homology to the TMPs of select prophages and cultured phages. The nearest neighbors in Genbank were TMPs encoded by *B. eiseniae* (35% similarity) and *B. licheniformis* (34% similarity) prophages and the *B. megaterium* phages Staley, Stahl, Stills and Slash (~ 32% similarity).

Basilisk gp47 was previously found to contain multiple conserved domains: an N-terminal phage related minor tail protein domain (PhageMin_T) that is overlapped by a conserved tape measure protein domain (TP901), a LysM-containing murein endopeptidase MepM/NlpD domain that overlaps a peptidase M23 domain (NlpD), and a third domain with homology to conserved domains of multiple coiled-coil proteins including neuromodulin-N, SMC, and DUF342 (Additional file 4: Figure S4A). Phylogenetic analyses of the N-terminal tape measure protein domain (TP901) and flanking sequence corresponding to gp47 residues 1–775 support a robust evolutionary relationship between the BLP TMPs and near neighbors including *B. eiseniae* and *B. licheniformis* and the four *B. megaterium* phages, Staley, Stahl, Stills, and Slash (Fig. 5b).

The presence and organization of conserved domain families within in the TMPs of v_B-Bak1, v_B-Bak6, and v_B-Bak10 and PBC4 is similar with select distinctions present among the three major domain regions (Additional file 4: Figure S4A box 1–3). The N-terminal minor tail protein domain (Additional file 4: Figure S4A, Box 1, residues 337–677) is similar among the BLPs and also appears broadly conserved among *Bacillus* phage TMPs (Additional file 4: Figure S4). While the three newly characterized phages and Basilisk possess a single N-terminal TMP domain (TP901) the corresponding region in PBC4 displays a more complex domain architecture (Additional file 4: Figure S4a). PBC4 orf17 possesses the TMP domain as well as a more extended region of homology to the phage-related tail protein domain family (COG5283) which encompasses multiple additional conserved domain families, including an additional tail-related domain at the proximal N-terminus, a Major Facilitator Superfamily (MFS-1) domain adjacent to a neuromodulin domain (PTYZ00121) as well as a Type I restriction modification system domain (COG0610) (Additional file 4: Figure S4a).

In addition, the PBC4 TMP possesses an insertion of 64 amino acids at residue 1591 positioned between the peptidase domain and C-terminal domain. In contrast to our phages and Basilisk, the neuromodulin domain (PTZ00121) in PBC4 orf17 is found in the N-terminal region of the protein downstream and adjacent to the TMP domain. A similar domain architecture is present in the prophage homologs encoded by the *B. eiseniae* and *B. licheniformis* (Additional file 4: Figure S4A). A low homology region upstream of the conserved peptidase domain in the central region of the TMP distinguished the BLPs from other putative homologs in Genbank (amino acid residues 1377–1509) (Additional file 4: Figure S4A).

The BLP TMPs each possess an M23 peptidase domain (pfam01551) (Additional file 4: Figure S4A Box 2). M23 peptidase family members are zinc-metallopeptidases belonging to the Gly-Gly endopeptidases [20]. The BLP TMP endopeptidase domains appear more closely related to host proteins than phage/prophage sequences. Comparison of the corresponding region in the TMPs of the *B. eiseniae* and *B. licheniformis* prophage homologs and the *B. megaterium* phages reveals two small domains in this region including a lytic transglycosylase lysozyme domain and a small LysM domain that may function in binding and cleaving host peptidoglycan (Additional file 4: Figure S4A). The C-terminal domain previously annotated as DUF382 in Basilisk gp47 (Grose 2014a) was present in the TMP of our phages and displays homology to multiple conserved domain families of proteins possessing coiled-coil motifs, including the neuromodulin-N and KELK (COG1340) superfamilies, the SMC-N superfamily and Myosin domains (Additional file 4: Figure S4B). Coiled-coil motifs are known to mediate protein-protein interactions and thus we speculate that this domain may be involved in interacting with potential TMP binding partners. Previous studies have identified putative binding partners for phage TMPs including assembly chaperones, mycobacteriophage proteins with domains that have sequence identity to Rpf family domain proteins that are hydrolases and have been associated with re-activating dormant cells and, lastly, domains that interact with superinfection exclusion proteins [15, 21–25]. The NB094/SMC_N domain present in the c -terminal region of the PBC4 TMP is also present in other related homologs, with the exception of phages Stills, Basilisk and our phages. The conserved domains in the PBC4 C-terminus include domains known to form multimers, bind ATP (NBD94 and SMC_N domains) and function in protein polymerization (spectrin and Ezra domains).

Similar to other dsDNA siphophages, we find the BLP TMPs possess hydrophobic regions (amino acids 624-757aa) predicted to form transmembrane or membrane-associated domains (Additional file 5: Figure S9) [26]. In silico transmembrane predictions indicate four putative transmembrane regions spanning the predicted MFS-1 domain in PBC4 and the corresponding positions in our phages and Basilisk (Additional file 5: Figure S9, A and B). Recent data suggests these TMP motifs may participate in chaperone recruitment needed for higher order interaction in tail assembly [15, 21, 26].

### Tail fiber-associated proteins

Two alternate tail fiber proteins are annotated within the virion modules of Basilisk and PBC4 although their designated open reading frames differ. Basilisk proteins gp49 (minor structural protein) and gp51 (hypothetical protein) correspond to PBC4 orf19 (putative tail fiber protein) and orf21 (receptor binding protein) and share sequence similarity and position with the putative virion modules of our phages (Fig. 3b). Both proteins exhibit homology to annotated TFPs of related phages. Basilisk gp49 was previously shown to exhibit sequence homology to the TFPs encoded by phages Slash and Staley [8]. The TFPs encoded by our phages, Basilisk (gp49), and PBC4 (orf19) share 67% amino acid similarity to the TFPs of phages Staley, Stahl, Stills, Slash (N-terminal, 79% coverage) and 64% to *B. licheniformis* prophages (N-terminal sequence, 54% coverage). The C-terminal ~ 150 amino acids exhibit limited homology to minor structural proteins and hypothetical proteins of a broad number of *B. cereus* phages. PBC4 orf19 (TFP) was found to uniquely encode an N-terminal sspH domain (residues 290–348) common to acid-soluble spore protein (SASP) type H. Proteins with sspH domains have been shown to impact sporulation of *Bacillus spp.*

The TFPs of our phages and Basilisk (gp51) share 96.5% percent amino acid similarity and exhibit low similarity to homologs in Genbank including a *B. gaemokensis* hypothetical protein (54%), proteins in phages JL, Shanette (both 52%), and CP-51 (51%), and a carbohydrate binding protein encoded by *B. cereus* (58% similarity, 23% coverage)*.* The conserved C-terminal region is highly diverse with multiple SNPs and amino acid variations and possesses an interleukin-like epithelial-to-mesenchymal-transition (EMT) inducer (ILEI) family domain sequence present in the BLP TFPs (Basilisk gp51, PBC4 orf21) (Additional file 6: Figure S8A and B). The PBC4 TFP shows 68% similarity in amino acid sequence to homologs in our phages and Basilisk and is 142 amino acid residues longer (Additional file 6: Figure S8C). In addition, the PBC4 TFP uniquely possesses a peptidase F family domain (COG1164) in the central region of the protein that is weakly homologous to structural proteins broadly conserved among other *Bacillus* phages.

Basilisk gp52, annotated as a hypothetical protein, lies immediately downstream and adjacent to the TFP within the virion module (Fig. 3b). The gp52 product was previously speculated to facilitate host cell access based on similarity to a *Paenibacillus chitinase* [8]. Each of our phages possess gp52 homologs at the same locus and exhibit high sequence similarity with the Basilisk homolog (Fig. 3b and c). A 1.5 KB region of nucleic acid sequence variation spans the C-terminal region of gp51 and the N-terminal region of gp52 resulting in multiple instances of non-synonymous amino acid variation in both proteins. While a gp52 homolog is absent in PBC4, a *B. cereus* phage PBC2 homolog (orf199, AKQ08514.1) represented the only significant homolog following BLASTP analysis.

### Putative capsid

Phage Basilisk was previously shown to possess a *T* = 9 icosahedral capsid [6] with architectural attributes consistent with the HK97-fold family, despite lack of significant sequence homology to known capsid structural domains. Our phages contained homologs to the hypothetical genes encoding the putative major capsid protein (MCP) of Basilisk gp36 and PBC4 orf 006 with high amino acid similarity > 99% to Basilisk gp36. The putative MCP is located downstream of the terminase gene (Basilisk gp32) with three intervening hypothetical proteins (gp33–35) that displays no significant homology to portal, minor head, or proteases often associated with capsid morphogenesis. Updated BLASTP analysis indicates the putative MCP of our phages and Basilisk possess moderate similarity to putative homologs encoded by Stills (81%), Staley (81%), and *B. licheniformis* prophages (73%).

### Lytic module

Phage-encoded lytic modules contain distinct classes of diverse peptidoglycan hydrolases (PGHs) including disruptive muralytic enzymes and holins that mediate degradation of the cell wall and membrane. The genomes of phages Basilisk and PBC4 each encode a two-component lytic cassette comprising a N-acetylmuramoyl-L-alanine amidase (MurNAc-LAA)-type autolysin homolog and a holin, corresponding to Basilisk gp54 and gp55 and PBC4 orf25 and orf26, respectively [8, 9]. Bacterial MurNAc-LAA peptidoglycan hydrolases hydrolyze amide bonds containing N-acetylmuramoyl and L-amino acids in the peptidoglycan cell wall.

Gram-positive autolysins are frequently modular proteins containing an N-terminal catalytic module and a C-terminal cell wall binding domain [30]. The BLPs endolysins possess an N-terminal amidase_3 domain whose enzymatic activity was experimentally verified for PBC4 (lysPBC4), and a putative C-terminal cell wall binding amidase02.C domain separated by a putative random coil linker [8, 9] (Additional file 6: Figure S8D and E). The C-terminal amidase_02C domain was previously predicted to be a novel cell-wall binding domain based on its similarity to the functional amidase_02C domain of the *B. anthracis* phage BtCS33 endolysin [9, 31]. The lysin homologs of our phages display complete identity with Basilisk gp54 and 63.4% similarity to the PBC4 homolog (LysPBC4). Domain-specific comparisons revealed the PBC4 homolog exhibited 69% nucleic acid sequence identity with putative endolysin of our phages and Basilisk at the N-terminal amidase_3 domain and only 33% at the C-terminal amidase_02C domain (9, present data).

The closest relative of Basilisk gp54 was previously reported to be the endolysin homolog of siphophage Staley [8] while the closest relatives of lysPBC4 were reported to be bacterial autolysins [9]. We performed updated BLASTP comparisons to gain further insight into their potential evolutionary homologs. The endolysin encoded by our phages and Basilisk shows closer similarity (86%) to the host-encoded cell wall hydrolases (endolysins) of five select strains of *B. cereus*, *B. anthracis, and Bacillus sp. N35–10-2.* Broad homology was also observed across a plethora of endolysins encoded by *B. cereus*, *B. wiedmannii*, *B. thuringiensis,* and *B. anthracis* (~ 85%) as well as an experimentally verified prophage-derived autolysin (AmiBA2446) from *B. anthracis* [32]. Homology to the near-full length gp54 (~ 99% coverage) was observed only for the seven phages vB_BtS_BMBtp14 (68%), PBC4 (lysPBC4) (63%), AP50 (59%), *B. cereus* phage PBC1 (57%) and phage 128,126 (55%). Homologs of seven phages exhibited greater homology (88% similarity) to the MurNAc-LAA domain spanning the N-terminal and central region of the gp54 homolog (Additional file 6: Figure S8D), including BCP78, JBP901, PBC6, BM10, TsarBomba, 3QCM8, and Bc431v3. Updated BLASTP analysis of lysPBC4 revealed lower overall similarity (< 72%) to both host and phage endolysin homologs. Only two phages currently in Genbank shows near full-length homology to lysPBC4: phage vB_BtS_BMBtp14 (72% similarity) and Basilisk gp54 (63% similarity). The N-terminal MurNAc-LAA domain of lysPBC4 (Additional file 6: Figure S8E), shows approximately 66% similarity to the MurNAc-LAA domains encoded by eight other *Bacillus* phages. In our analysis, Basilisk gp54 and its homologs in our phages show no homology to the endolysins encoded by *B. megaterium* J group phages.

### Holin

Phage-encoded holins are temporally-regulated membrane proteins that maximize optimal phage burst times [33]. The holin homologs encoded by our phages share complete identity with the Basilisk phage holin (gp55) and 92% similarity to the PBC4 holin (orf026). The BLP holins possess a putative conserved holin-SPP1 domain. SPP-1 family holins encoded by other *Bacillus* phages and prophages exhibit a broad gradient of amino acid sequence similarity in Genbank, with limited division in percentage similarities. In contrast, the Basilisk-like phage holins show no close relatives in Genbank and exhibit < 50% similarity to select *Bacillus* prophage holins including *Parageobacillus* sp. (~ 49%) and *B. okhensis* (47%), *B. licheniformis* (40%), and diverse members of the *B. cereus* group (37–41%). SPP1 family holins typically comprise two putative helical transmembrane (TM) domains at the N-terminus separated by a short beta-turn domain [34] and possess a charged, polar C-terminal domain. The presence of two N-terminal transmembrane (TM) domains in the BLP holin sequences at shared positions (Additional file 7: Figure S10) was verified using the ExPASy TMHMM program (http://us.expasy.org/). In the BLPs, discriminatory non-synonymous variation is localized specifically to the C-terminal domain at positions 62, 71, and 74.

### DNA metabolism genes

#### tRNAs

Both Basilisk and PBC4 were previously reported to encode two tRNA genes at similar positions in the genome [8, 9]. The tRNA^Trp^ was present in both PBC4 and Basilisk while the second tRNA differed between the two phages (Basilisk = tRNA^Asn^, PBC4 = tRNA^Asp^). Our phages possess the two tRNA genes shared by Basilisk (Additional file 8: Figure S5). Updated tRNA homology searches using tRNAscan-SE [37] revealed our phages and Basilisk also uniquely encode tRNA^SeC^ gene downstream of the tRNA^Trp^ and tRNA^Asn^ genes Basilisk (Additional file 8: Figure S5). This rare tRNA gene is central to selenoprotein biosynthesis (reviewed in [38]) and is, to our knowledge, the first example of such a gene identified in bacteriophages. In addition, phage v_B-Bak10 uniquely encoded a third tRNA gene (tRNA^Ala^) downstream of the tRNA^Trp^ and tRNA^Asn^ genes.

### tRNA synthetase-like domain protein

Our analysis of the v_B-Bak10 phage genome (Fig. 5) revealed the presence of a novel open reading frame (orf60) spanning the position of Basilisk gp58 that displays 76% amino acid similarity (77% coverage) to a novel hypothetical protein encoded by two *B. cereus* strains, BAG1X2–2 (EOO44202.1) and BAG2O-1 (EOP00399.1) (Fig. 3c). The two *B. cereus* homologs are 42 residues in length and possess a conserved bacterial class I K lysyl-tRNA synthetases (PFAM01921) domain that maps to the canonical connective protein (CP) domain of the reference protein (also known as the core domain) (Additional file 9: Figure S6A-C). The phage v_B-Bak10 and *B. cereus* CP proteins share major secondary structure characteristics with the ARS CP domain regions from taxonomically diverse bacteria, including three flexibility regions and major beta strand placement, and antigenicity index plots (data not shown). Aminoacyl-tRNA synthetases (ARSs) are central to the process of protein translation functioning to join tRNAs to their cognate amino acids with high specificity while ensuring translational fidelity by removing mis-activated amino acids [41]. The CP domain of the ARS plays a central role in determining the specificity and efficiency of aminoacylation [42]. The putative ARS CP-domain homologs within the genomes of *B. cereus* strains BAG1X2–2 and BAG2O-1 are located in DNA metabolism islands downstream of a suite of tRNA genes and upstream of a putative prophage (data not shown). In the v_B-Bak10 phage the gene is located at the left most boundary of the DNA metabolism module upstream of the holiday junction resolvase (HJ) and DNA metabolism genes (Fig. 3c, DR1). Greater inspection of the genes at this boundary revealed that the hypothetical gene encoding Basilisk gp60 and its homologues in v_B-Bak1 and v_B-Bak6 (Fig. 3c, DR1) exhibit 74% similarity to hypothetical protein IGA_05652 of *B. cereus* HuA3–9 and 51% similarity (74% coverage) to valine-tRNA synthetase (ligase) of *Leptospirillum sp.*

### Phosphoadenosine phosphosulfate reductase (PAPS reductase/CysH)

The Basilisk phage genome encodes a PAPS reductase homolog (gp11) [8]. Among our phages, only v_B-Bak10 was found to encode a PAPS reductase (99% aa identity, Fig. 3a). The gene is absent in PBC4. The v_B-Bak10 PAPS reductase gene resides in the same physical location as Basilisk gp11 in the left terminal region of the genome. The closest relatives of the PAPS reductase homolog of v_B-Bak10 and Basilisk were phage-encoded PAPS homologs from phage MG-B1(67%), phage Eldridge (64%) and prophage homologs from *B. thuringiensis* (57%), *B. cereus* (56%) and *Paenibacillus* species (54%). PAPS-reductases (CysH in bacteria) function in the reduction of 3′– phosphoadenylylsulfate (PAPS) to phosphoadenosine-phosphate (PAP) using thioredoxin as an electron donor. Phage PAPS reductases are members of the adenine nucleotide alpha hydrolase superfamily (cl00292) and are thought to impart selective advantages to their host by facilitating inorganic sulphate assimilation [45]. PAPS reductases are relatively rare in phages [46], however knowledge of their prevalence in bacteriophages is increasing as genome sequences continue to accumulate. Genbank homology searches revealed that, to date, only 11 phage genomes encode a PAPS reductase homologs, including eight *Bacillus* phages (including Basilisk), *Clostridium* phage phiCP13O, *Mycobacterium* phage Gaia, and *Streptococcus* phage D4276.

### Terminase

The terminase genes of phage v_B-Bak10 and Basilisk (gp32) displays 99% nucleic acid identity and were distinguished from the terminase gene of phages v_B-Bak1 and v_B-Bak6 by the presence of 18 shared synonymous SNPs. BLASTP homology analysis of the terminase gene sequences for phages v_B-Bak1, v_B-Bak6, v_B-Bak10 revealed moderate similarity to only those encoded by phages Stills (70%), Slash (69%), Stahl (70%) and Staley (60%). The Basilisk terminase gene orf32 and the adjacent upstream gene encoding a hypothetical protein (gp31) shared distinguishing SNPs with v_B-Bak10.

### Poly [ADP-ribose] polymerase (PARP) domain protein

Within the replication the module of these phages (44.2 kb–50.2 kb) v_B-Bak10 uniquely encodes an 37-residue hypothetical protein that possesses a conserved poly [ADP-ribose] polymerase (PARP-reg) family domain (PLN03123) spanning 76% of the length of the putative product. The gene is positioned adjacent to the recB exonuclease gp68 and is collinear with Basilisk gp69 (hypothetical protein) (Fig. 3c, DR2). The amino acid sequence displays 47–52% similarity to PARP domains of the poly [ADP-ribose] polymerase 1 proteins of plant origin. PARP domain containing proteins function in DNA repair and facilitate genome integrity. PARP family proteins are evolutionarily ancient and ubiquitous across the domain of life (with the exception of *Archae* and *S. cerevisae*) [51]. Our BLASTP domain searches revealed no PARP domain proteins in other *Bacillus* phages.

### Ribonucleotide reductases (RNRs)

The Basilisk and PBC4 phages each encode ribonucleotide reductases (RNRs) with minor differences in their respective annotations [6, 9]. The Basilisk phage genome annotation specifies two genes, gp91 and gp92, as homologs of microbial NrdE (flavodoxin protein) and NrdI (NrdE stimulatory protein) that corresponds to PBC4 ORFs 57 (hypothetical protein) and 58 (putative RNR). In our analysis, PBC4 orf56 (putative RNR) and Basilisk gp90 (hypothetical protein) both display homology to class Ib NrdF. Comparative genomic analysis revealed the genomes of our phages also encode a collinear NrdF-NrdE-NrdI cassette as well as a distally-located NrdH homolog. The single glutaredoxin-like NrdH homolog is present approximately ~ 51 kb upstream of the RNR gene cluster in the left terminal region of the genomes of our phages, Basilisk (gp14) and PBC4 (orf112) (Fig. 3a). The NrdFEI homologs of phages v_B-Bak1 and v_B-Bak6 exhibit near identity to the corresponding homologs of Basilisk RNRs while the NrdH homologs of our three phages are identical and genetically distinct from the Basilisk and PBC4 homologs. The Basilisk and PBC4 NrdF homologs gp90 and orf56, respectively, share 92% amino acid similarity. The NrdF homologs of phages v_B-Bak6 and v_B-Bak1 were highly similar to Basilisk gp90 (99%) and PBC4 orf56 (93%) while the v_B-Bak10 homolog exhibited 96% and 91% similarity, respectively. Similar to NrdF, the NrdE and NrdI homologs of phages v_B-Bak1 and v_B-Bak6 were also highly similar to the respective Basilisk homologs (99–100%) with less similarity observed between these homologs and PBC4 orfs 57 and 58. The v_B-Bak10 NrdE homolog was 93% similar to Basilisk gp91 and 88% similar to PBC4 orf57.

BLASTP analysis revealed the closest relatives to the BLP NrdFEI homologs were prophage-derived homologs encoded by diverse *Paenibacillus* and *B. cereus* group representatives (~ 55%) with greater homology noted for NrdE (~ 58%) than NrdF (~ 50%) while NrdI was intermediate (~ 55%). The NrdH homologs of our phages are identical in sequence and display 93% amino acid similarity to Basilisk and lesser similarity to PBC4 (42%). The NrdH homologs exhibited < 35% similarity to homologs encoded by *Bacillus spp., Mycobacterium spp* and other NrdH representatives (> 69 residues). PBC4 orf112 displays up to 46–48% identity to homologs within *Mycobacterium abscessus* sp. and ~ 40% to the NrdH-redoxins of multiple *Firmicutes*.

### Integrase

Phages Basilisk and PBC4 were previously found to encode XerC/XerD site-specific recombinases [8, 9]. The v_B-Bak10 integrase homolog (Fig. 3d) was identical to Basilisk gp109 and shows 85% similarity to homologs encoded by PBC4, and lesser similarity to phages Stahl (42%), Stills (40%), Staley (40%), Slash (40%) and *B. licheniformis* prophage homologs (34%). The v_B-Bak1 and v_B-Bak6 integrase homologs are identical in DNA and amino acid sequence and display 99% similarity in amino acid sequence (95% DNA identity) to the v_B-Bak10 and Basilisk homologs. The v_B-Bak1, v_B-Bak6, v_B-Bak10 integrase homologs display a similar level of homology to the integrase homologs present in other J1 group phages and *B. licheniformis*.

### Virulence genes

#### DUTPase

Genes encoding dUTPases were previously reported in the Basilisk phage genome (gp21) and PbC4 (orf119) [6]. Homologs of the putative Basilisk and PBC4 dUTPases were also encoded by our phages (Fig. 3a). Viral dUTPases function in pyrimidine biosynthesis and support genome integrity by lowering intracellular dUTP concentration thereby preventing its incorporation into the genome. The v_B-Bak1 and v_B-Bak6 dUTPase homologs show 98% similarity to the phage Basilisk dUTPase (gp21) while the v_B-Bak10 homolog displays lower similarity (86%). The dUTPases of Basilisk and PBC4 homologs display only 67% similarity. The central region of the dUTPase protein exhibits elevated diversity (Additional file 10: Figure S7) and appears unique to the BLP homologs based on our BLASTP searches. The N-terminal region displays limited homology to chromosomal *B. cereus* homologs (44%) while the conserved dUTPase domain (PFAM08761) at the C-terminus shows 67% similarity to the dUTPase of *B. licheniformis sp*.

#### PhoH

Our phages encoded homologs of PhoH genes previously found in the Basilisk (gp85) and PBC4 (orf052) (Fig. 3c, DR3). In bacteria, the Pho regulon functions in phosphate regulation that promotes survival under conditions of phosphate starvation and putative homologs are rare in non-marine phages [6, 59]. Amino acid sequences of v_B-Bak1 and v_B-Bak6 PhoH homologs were identical to Basilisk gp85 while the v_B-Bak10 homolog exhibits intermediate similarity (97%) between PBC4 and v_B-Bak1, v_B-Bak6, and Basilisk homologs. BLASTP analysis revealed low-level homology to two similar groups of bacterial and phage homologs. The PhoH homologs of v_B-Bak61, v_B-Bak6 and Basilisk exhibited 52% similarity to homologs encoded by Staph phage vb_SscM-1 (52%), *B. andreroultii* (52%) and *B. coagulans* (51%). A second similarity group of homologs encoded by phages JBP901, PK16, BM10, Bcp1 and vb_Bcem_Bc431v3 exhibited less similarity (41–43%).

#### Gene expression

Our phages also encode two putative transcription factors previously identified in the genomes of Basilisk (gp22/gp103) and PBC4 (orf120/orf066) (Fig. 3a). Basilisk gp22 was previously shown to possess a conserved rsfA-family transcriptional regulator domain [8]. RsfA is a putative prespore-specific regulatory gene [60], and is widely prevalent in *Bacillus* species. The Basilisk gp22 homolog in our phages show 99% identity to the Basilisk product and 91% to the PBC4 homolog. The BLP rsfA homologs appear novel with only extensive low-homology (< 40% similarity) hits among diverse *Bacillus* species in Genbank. The second putative transcription factor (Basilisk gp103, PBC4 orf066) possesses an N-terminal phage regulatory protein Rha domain (PFAM09669) and a C-terminal phage antirepressor protein KilA-C_ANT (antirepressor) superfamily domain [8]. KilA proteins possess conserved N-terminal (KilA-N) and C-terminal (KilA-C) domains. Both domain families are putatively involved in DNA-binding.

BLASTP comparisons indicated the N-terminal Rha domain homologs of v_B-Bak1, v_B-Bak6, Basilisk and PBC4 exhibited significant homology to *B. cereus sp*. homologs (97% similarity). In contrast, the v_B-Bak10 homolog appears more diverse displaying lower intragroup similarity to the BLPs (~ 83%) and only 78% and 70% similarity observed to Rha domains of select *B. cereus* strains and multi-species *Bacillus* members, respectively. Overall, the conserved Rha domain exhibited little diversity while the C-terminal KilA-C domains appear highly diverse in both sequence and predicted secondary structure (data not shown). The related J group *B. megaterium* phages Stahl, Slash, Staley also encode homologs of Basilisk gp103 that possess both an N-terminal Rha regulatory domain and a C-terminal KilA-C_ANT domain with ~ 59% overall similarity to the BLP homologs. In contrast, phage Stills possesses an N-terminal Bro domain in place of the Rha domain. Previous studies have also identified the Rha domain inhabiting the same proteins in similar orientation as Bro-N domains [61]. A Bro-N family domain was identified in a hypothetical protein encoded by both v_B-Bak10 and PBC4 (orf081) that display ~ 73% similarity to Basilisk gp117 and homologs encoded by phages v_B-Bak1 and v_B-Bak6. Homology searches did not reveal a Bro-N family domain in Basilisk gp117 or the v_B-Bak1 and v_B-Bak6 homologs despite sequence similarity to Bro-toxin proteins encoded various *Bacillus* organisms (51–72%).

### RNApol sigma-70 factor family protein

The BLPs each possess a hypothetical protein containing a conserved RNA polymerase sigma factor 70 domain. The BLP sigma70 homologs possess approximately 91% intragroup amino acid sequence similarity. The closest neighbors of the PBC4 sigma70 homolog were putative homologs encoded *B. licheniformis* sp. (64% similarity) and the J group of *B. megaterium* phages Stills, Stahl, Staley, and Slash (~ 60% similarity). Similar yet marginally lower similarity was evident for Basilisk and our phages (*B. licheniformis*-61%, J group phages- ~ 59%). The sigma 70 homologs in each of the BLPs lie adjacent and upstream to the putative dUTPase (Basilisk gp21) and RsfA transcriptional regulator (gp22) and are flanked by hypothetical genes (Fig. 3a). The respective homologs in our phages and Basilisk (gp020) exhibit similar lengths, however homology to the sigma70 domain is limited to a region spanning N-terminal residues (78 aa, residues 157–235). The conserved sigma70 domain of PBC4 (orf118, 236aa) spans the central and C-terminal region of the protein (157aa, residues 76–233) and possesses the highly conserved promoter recognition helix as well as the RNApol binding determinant (data not shown). The reduced homology to the sigma70 domain in our phages and Basilisk was due to amino acid variation in the central region of the protein. This sequence diversity imparts alterations in the secondary structure of the sigma70 core domain region responsible for promoter recognition.

## Discussion

### Host range

The biological basis for the observed host specificity for *B. anthracis* exhibited by v_B-Bak1, v_B-Bak6, and v_B-Bak10 phages here remains unknown and is a subject of our ongoing research. Previously, the Basilisk phage was reported to infect the *B. anthracis* Sterne vaccine strain [8], however, its ability to infect diverse wild-type *B. anthracis* isolates remains untested. In addition, cross-infectivity of PBC4 for *B. anthracis* was not assessed [9]. It is possible both Basilisk and PBC4 may possess activity for *B. anthracis* similar to our phages though this remains undetermined. Our phages exhibit greater overall specificity within the *B. cereus* group compared to phage Basilisk.

### Structural module

The observed variation in virion tail lengths of our phages do not correspond to TMP gene size. Tape measure proteins are ubiquitous in tailed phage genomes, although the specific mechanistic attributes of tail length determination for many phages remain largely uncharacterized. For some phages, this process involves protein-protein interactions that may indirectly impact tail length such as phage-encoded chaperones that experience post-translational processing [15]. It is possible that TMP-associated proteins and hypothetical proteins could play some role in generating morphological variation in addition to that associated with the nascent lengths of encoded TMPs genes. The C-terminal variation observed in the TFPs is consistent with other phage TFPs where the structural domain is more conserved while the C-terminus exhibits greater variability due to potential ligand interactions that impact fitness and mediate host specificity (Lingohr 2008 [27]). Tail fiber gene diversity between Basilisk (gp49) and PBC4 (gp19) was previously speculated to contribute to the observed host range differences or variable lytic phenotypes [9]. Whether the observed sequence differences in the putative TFPs encoded by our phages play any role in specificity to *B. anthracis* remains to be tested.

Although gp52 was previously speculated to be a putative chitinase [8], in our analysis, we find this similarity is weak (28% identity, over 248aa) and limited exclusively to a C-terminal FNIII domain common to a diverse array of known prokaryotic and cellular FNIII-domain-containing proteins. Together, the homologs lacked detectable similarity to enzymatic chitinases domains. With the exception of the putative FNIII domain, PFAM/Genbank searches revealed no other conserved domain families are present based. The C-terminal domain of select prophage and phage host recognition proteins that form the tail tip and central tail fiber structures have previously been shown to contain FNIII domains, including the gpJ protein of phage Lamda and pb3 protein of phage T5 [28, 29]. We speculate gp52 is a structural tail-associated protein.

### Lytic module

Full-length sequences of the putative BLP endolysins show closer overall homology to *Bacillus* group members than cultured phages. We speculate this pattern may reflect gene acquisition from host *Firmicutes*. Other authors have previously speculated that instances of strong homology observed between *B. cereus* phage MurNAc-LAA endolysins and host autolysins may reflect horizontal transfer between *Bacillus* phages and various *B. cereus* group hosts [30]. The molecular diversity of the BLP lysins and their homologs is exhibited primarily by the presence or absence of the C-terminal amidase_02C domain. Consistent with previous data on lysPBC4 [9] the N-terminal amidase_3 domain of the BLPs show greater conservation among their homologs than the C-terminal amidase_02C domain and linker region that are absent in most phage endolysin homologs. Full-length sequences of the putative BLP endolysins display closer overall homology to *Bacillus* group members than cultured phages. We speculate this pattern may reflect gene acquisition from host *Firmicutes*. Other authors have previously speculated that instances of strong homology observed between *B. cereus* phage MurNAc-LAA endolysins and host autolysins may reflect horizontal transfer between *Bacillus* phages and various *B. cereus* group hosts [30]. The molecular diversity of the BLP lysins and their homologs is exhibited primarily by the presence or absence of the C-terminal amidase_02C domain. Consistent with previous data on lysPBC4 [9] the N-terminal amidase_3 domain of the BLPs show greater conservation among their homologs than the C-terminal amidase_02C domain and linker region that are absent in most phage endolysin homologs. The charged basic C-terminal domain is considered the most highly-conserved feature of SPP1 holin family [33] and may experience strong evolutionary selection to achieve optimal host or niche-specific lysis times [33, 35, 36]. Mutational plasticity within this polar C-terminal domain is thought to mediate regulatory functions at the cytosolic surface of the protein whereby alteration in the number of positively charged residues may modulate the lytic clock [33].

### tRNAs and tRNA synthetase gene

Phages are generally considered to evolve towards optimal synergy with the host translational machinery via translational selection for host-preferred codons. Acquisition of select tRNAs may compensate for overused codons in the phage genome thereby maintaining translational efficiency of phage genes while reducing the impetus to co-evolve host codon usage patterns [39, 40]. The unique tRNA composition the BLPs could be due to translational bias between their natural host species. The biological significance of the putative gene tRNA^SeC^ is unknown. Although a plethora of functional yet truncated ARS paralogs and pseudogenes have been observed in bacterial genomes [43], to our knowledge, no such truncated ARSs have yet been reported in phage genomes. With the exception of tRNA genes, the overall presence of translation-associated genes in phages is rare [39] although select myoviruses and mimiviruses are known to encode complete ARS genes [43, 44].

### PAPs

In some *B. cereus* lineages, the CysH (bacterial PAPS) was identified as a target of potential ecological specialization or adaptation to host environmental requisites [47]. As part of the cysteine biosynthesis gene cluster (cysH-ylnABCDEF) in *Bacillus sp.,* CysH is important in mediating biosynthesis of cysteine from sulfate. Regulation of this system has recently been linked to the PlcR virulence regulon and the oxidative stress response [48]. CysH has also been suggested to be indirectly involved in pathogen virulence in scenarios where sulfur limitation alters the expression of known host virulence determinants, such as siderophore pyochelin biosynthesis in *Burkholderia* [45], survival and virulence in *P. aeruginosa* biofilms [49] and activity as potential global signal regulators in *E. coli* [50]. Future experimental studies are warranted to test the impact of phage-derived CysH homologs on phage-host interactions and host phenotype.

### Ribonucleotide reductases (RNRs)

The BLPs encode a complete Type Ib suite comprising a NrdI-NrdE-NrdF cassette in a sequential order that is shared with many microbial representatives [54]. For *B. anthracis,* class Ib RNRs are thought to play an important role in life cycle of the pathogen, facilitating spore germination and modulating proliferation and survival through promoting rapid nucleotide synthesis needed to facilitates vegetative proliferation during infection [55]. Similar to *B. anthracis,* the NrdH of the BLPs is not co-located with the NrdIEF cassette. The *B. anthracis* nrdE gene also contains a phage-like group I intron generating two exons for this gene [56]. Phage-derived RNRs have previously been proposed to modulate alterations in host DNA metabolism [57]. The RNR genes of *Bacillus* prophages are also targets of parasitic genetic elements [58] and have been associated with targets of phage resistance. RNRs are highly represented in cultured and environmental viral metagenomes and are found within all tailed dsDNA *Caudovirales* family members (*Podoviridae, Myoviridae, Siphoviridae*). RNRs are also highly conserved among *Bacillus* phages [8]. Recent genomic surveys suggests that although the *Siphoviridae* comprise the most represented group of sequenced phages, only 30% of those sampled possess RNRs [52]. It has been suggested phages may have acquired such Associated Metabolic Genes (AMGs) from their hosts as an evolutionary adaptation to their respective microenvironment [52]. Although the majority RNRs of *Siphoviridae* are predominantly class II (NrdJ) [52], the BLPs studied here possess a complete set of class Ib nrdHIEF genes. Class Ib and class III RNR operons, specifically, are highly conserved in *Bacillus spp.* hosts [53].

### Gene expression

Multidomain proteins with Rha, KilA-C, and Bro domains are broadly distributed in temperate bacteriophages, prophages, and large eukaryotic DNA viruses [61]. As putative phage antirepressors, KilA and Bro domain proteins have been suggested to function within the context of bacterial toxin-antitoxin systems (TAS) working in concert to regulate lytic gene expression [62]. Though Bro domains remain largely uncharacterized, their flexible domain architecture has been suggested to arise from combinatorial domain shuffling and posited to reflect the dynamic evolution of transcriptional regulation occurring in these viruses [61].

In bacteriophages, sigma70 proteins act as transcriptional switches mediating early-middle-late gene transcription [64]. The distinct predicted secondary structures discriminating the PBC4 sigma70 homolog from our phages and Basilisk lead us to speculate that the two variants may reflect unique requisites in promoter recognition. Bacterial hosts modulate sigma factor selection and transcriptional switching to adjust to environmental and metabolic stress, including starvation. For example, in *B. anthracis,* sigma70 expression is altered during transitions from vegetative growth to sporulation [63]. Bacteriophage-encoded transcriptional regulatory proteins have been shown to selectively displace host sigma70 from RNA polymerase (RNAP) and thereby alter host RNAP activity in favor of temporal regulation of phage gene expression (Brown 2016 [65]). Whether the putative sigm70-like proteins encoded by the BLPs and other J cluster phages function in a similar manner remains to be tested.

### Phage evolution

Unlike other evolutionary clusters, the J cluster *B. megaterium* phages Slash and Staley previously displayed minimal proteomic conservation with phage Basilisk (43.4%), also a J1 cluster member [6]. The present analysis incorporates our phages and PBC4, providing additional insight into the evolutionary structure of the J1 cluster phages. The BLPs share significant portions of their core and putative accessory genome modules with prophages from the evolutionarily divergent *Paenibacillus* genus, as well as *B. licheniformis* and *B. eiseniae spp*. These data provide further insight into potential evolutionary neighbors of the J1 cluster phages and suggest additional reservoirs of potential virulence genes. *Paenibacillus* genus members are large Gram-positive, endospore-forming bacteria that are evolutionarily divergent from the *Bacilli* while *B. licheniformis* is a ubiquitous soil bacterium utilized for agro-industrial protein production and diet supplementation of industrial swine. *B. eiseniae* is a novel Gram-negative halotolerant soil bacterium isolated from the intestinal flora of the earthworm *Eisenia fetida* and has been suggested to represent a potential microniche of *B. anthracis* [66]. The isolation sources of our phages were soil matrices taken from burial sites of anthrax-infected cattle. Such an environmental source could represent an environmental milieu favorable to the selective adaptation of these phages to *B. anthracis* hosts, although this remain purely speculative.

Multidomain diversity patterns in the TMP and KilA proteins of the J cluster phages illustrate potential instances of domain recombination. Mosaicism derived from gene-module exchange obfuscates estimates of phage population structure [67, 68]. Protein domain recombination (shuffling) represents an additional and largely uncharacterized layer of evolutionary and structural complexity of viral evolution [61, 69–71]. Mosaicism at the protein domain level illustrates the potential for phenotypic innovation by phage proteomes as diverse protein domain architectures are sampled within the microbial gene pool. Large-scale assessments of putative domain recombination among *Bacillus* phage proteins may yield additional insight into phage-host coevolution.

### Phylogeography and taxonomy

The BLPs are diverse in their geographical distribution, illustrated by their isolation sources in North America, Eurasia and East Asia. In contrast to their widely different geography, BLP representatives from North America (Basilisk) and the Caucasus region (Basilisk, v_B-Bak1, v_B-Bak6, and v_B-Bak10) appear genetically homogenous. Such pronounced genomic homogeneity has been observed previously among highly-related yet geographically-diverse phages [72, 73]. Global expansion of *Bacillus* host lineages encoding ancestral prophages is consistent with the broad geographical distribution of BLPs but does not account for the striking genetic homogeneity between our phages and Basilisk. While the basis of this trend unclear, it is possible that for the BLPs, such a dynamic may reflect coevolution with *Bacillus* hosts possessing conserved host range determinants. Though speculative, such factors could conceivably act as strong drivers of purifying selection.

Based on the high degree of genome-wide sequence similarity and gene conservation between our phages, Basilisk and PBC4, we propose that a new species called “Basilisk” be established within the *Siphoviridae* family encompassing our phages and Basilisk (> 95% identity), with the Basilisk phage representing the type species. In addition, we propose a new genus called “Basilisk-Like Phage” that includes both the proposed Basilisk species and phage PBC4 (> 80% identity). We use the term “BLPs” here informally in order to discuss evolutionary trends collectively among our three phages, Basilisk and PBC4.

## Conclusions

The genomes of the three novel *B. cereus* group phages reported here provide additional insight into the shared genomic architecture and molecular evolution of the J1 cluster phages reported by previous authors [8, 9]. To date, the three phages represent the only known close relatives of the Basilisk and PBC4 phages and their shared genetic attributes and unique host specificity for *B. anthracis* provides additional insight into candidate host range determinants.

## Additional files

Additional file 1: **Figure S1.** Geographical location of the Basilisk-like phages. A) global distribution of the Basilisk-like phages in North America, Eurasia/Caucasus, and East Asia., B) location of soil samples containing phages v_B-Bak1, v_B-Bak6, and v_B-Bak10 near the eastern city Kutaisi in the country of Georgia. Global base map obtained from http://www.ngdc.noaa.gov/, map of Georgia obtained from https://simple.wikipedia.org/ wiki/Georgia_(country)#/. (PDF 229 kb)

Additional file 2: **Figure S2.** Whole genome alignments of phage v_B-Bak1, v_B-Bak6, v_B-Bak10 and the Basilisk and PBC4 reference genomes. Nucleic acid sequence is designated by grey bars, SNP density is indicated by vertical black lines. Open Reading Frames (ORFs) are respective to the Basilisk phage genome are illustrated above the alignment for reference. (PDF 2006 kb)

Additional file 3: **Figure S3.** Whole-genome percent similarity among the phage genomes analyzed. (PDF 11 kb)

Additional file 4: **Figure S4.** Tape Measure Protein domain content and organization. (A) Physical locations of conserved family domains with the TMPs of the BLPs and near relatives. Boxes designate the three putative domain regions in the BLPs, including the N-terminal phage-related minor tail protein domain (box 1), peptidase domain (box 2), and C-terminal domains [3]. ^*a*^ phages v_B-Bak1, v_B-Bak6, v_B-Bak10 possess identical domain architectures, for brevity only v_B-Bak10 is shown here. Expanded views of all domain hits observed for v_B-Bak1, v_B-Bak6, v_B-Bak10, Basilisk are illustrated in (B) and phage PBC4 (C). (PDF 239 kb)

Additional file 5: **Figure S9.** Title of data: Predicted transmembrane helix domains (TMH) in the BLP TMPs Description of data: Predicted location and orientation of transmembrane helices in the TMP proteins of the BLPs, A) position of the predicted TMH domain, B) expanded view of (TMH) domain. (PDF 48 kb)

Additional file 6: **Figure S8.** Conserved family domains within the BLP TFPs and endolysin proteins. The positions of the conserved family domains within the TFPs of Basilisk, _B-Bak1, v_B-Bak6, v_B-Bak10 phages (A) and PBC4 (B) are illustrated along with the conserved family domains within the endolysins of Basilisk, _B-Bak1, v_B-Bak6, v_B-Bak10 phages (D) and PBC4 (E). (PDF 620 kb)

Additional file 7: **Figure S10.** Title of data: Predicated transmembrane helix domains (TMH) in BLP holin protein. Description of data: Predicted location and orientation of transmembrane helices in the holin proteins of the BLPs. (PDF 34 kb)

Additional file 8: **Figure S5.** Organization of putative tRNA genes encoded by the phages studied**. (PDF 8 kb)**

Additional file 9: **Figure S6.** Protein homology region of Aminoacyl-tRNA Synthetase (ARS) CP family domain encoded by phage v_B-Bak10. Crystal structure of the CP domain of bacterial ARS (A and B) and corresponding amino acid alignment of CP domain homologs (C). (PDF 722 kb)

Additional file 10: **Figure S7.** Amino acid sequence alignment of the putative DUTPase proteins encoded by the Basilisk-like phages. (PDF 3253 kb)

## Abbreviations

BLP: Basilisk-Like Phage
PAPS: Phosphoadenosine-phosphosulfate-reductase
RNR: Ribonucleotide Reductase
TFP: Tail Fiber Protein
TMP: Tape Measure Protein
