# Correction to: Genomic characterization of three novel Basilisk-like phages infecting *Bacillus anthracis*

**DOI:** 10.1186/s12864-018-5105-z

**Published:** 2018-09-27

**Authors:** J Farlow, D Bolkvadze, L Leshkasheli, I Kusradze, A Kotorashvili, N Kotaria, N Balarjishvili, L Kvachadze, M Nikolich, M Kutateladze

**Affiliations:** 1 George Eliava Institute for Bacteriophages, Microbiology and Virology, Tbilisi, Georgia; 2 Lugar Center for Public Health Research at National Center for Disease Contro, Tbilisi, Georgia; 3 Farlow Scientific Consulting Company, LLC, Lewiston, UT USA; 4 Department of Bacteriophage Therapeutics, Bacterial Diseases Branch, Walter Reed Army Institute of Research, Silver Spring, Silver Spring, MD USA

## Correction

Following the publication of this article [1], the authors noted two typographical errors: one in Table 1 with regard to the location of the Basilisk Phage, which was incorrectly captured as “Kutaisis, country of Georgia Utah, USA” but should be “Utah, USA”. The second error was an accidental tandem duplication of the following paragraph on page 14:

“Full-length sequences of the putative BLP endolysins display closer overall homology to *Bacillus* group members than cultured phages. We speculate this pattern may reflect gene acquisition from host *Firmicutes*. Other authors have previously speculated that instances of strong homology observed between *B. cereus* phage MurNAc-LAA endolysins and host autolysins may reflect horizontal transfer between *Bacillus* phages and various *B. cereus* group hosts [30]. The molecular diversity of the BLP lysins and their homologs is exhibited primarily by the presence or absence of the C-terminal amidase_02C domain. Consistent with previous data on lysPBC4 [9] the N-terminal amidase_3 domain of the BLPs show greater conservation among their homologs than the C-terminal amidase_02C domain and linker re- gion that are absent in most phage endolysin homologs.”

## References

[1] Farlow, et al. BMC Genomics. 2018;19(685). 10.1186/s12864-018-5056-4.

