# Supplementary figures and images for: Genomic characterization of three novel Basilisk-like phages infecting Bacillus anthracis
Source: BMC Genomics. 2018 Sep 18;19:685. doi: 10.1186/s12864-018-5056-4 (PMC6145125; doi:10.1186/s12864-018-5056-4)

A)

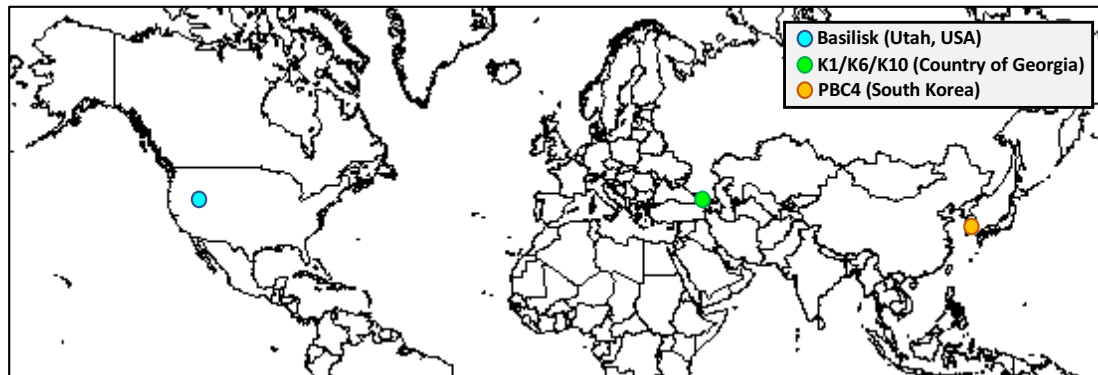

B)

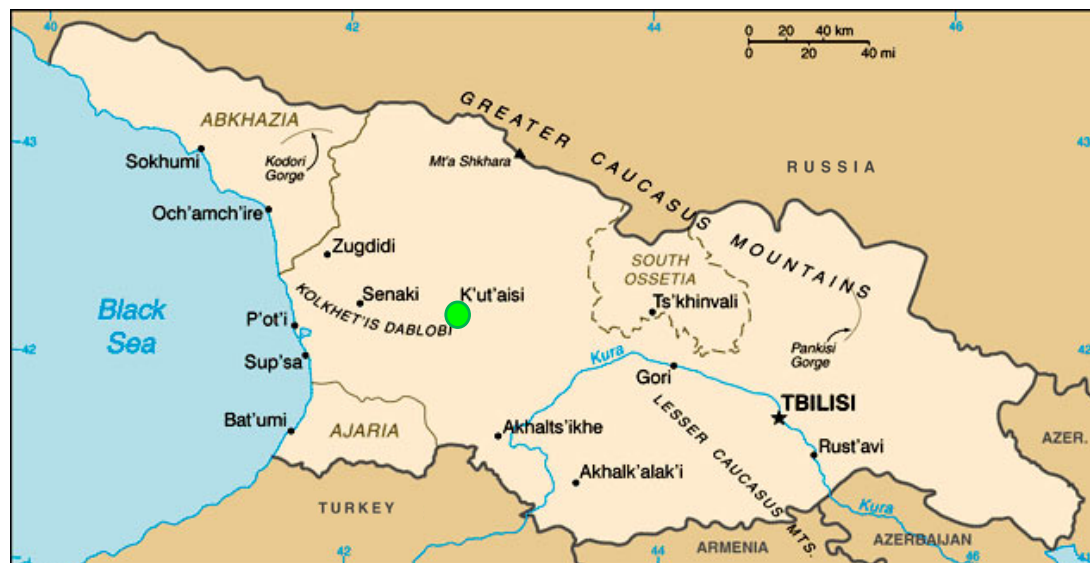

Supplement: Supplementary file 1 — Figure S1. Geographical location of the Basilisk-like phages. A) global distribution of the Basilisk-like phages in North America, Eurasia/Caucasus, and East Asia., B) location of soil samples containing phages v_B-Bak1, v_B-Bak6, and v_B-Bak10 near the eastern city Kutaisi in the country of Georgia. Global base map obtained from http://www.ngdc.noaa.gov/, map of Georgia obtained from https://simple.wikipedia.org/ wiki/Georgia_(country)#/. (PDF 229 kb) [file 12864_2018_5056_MOESM1_ESM.pdf]

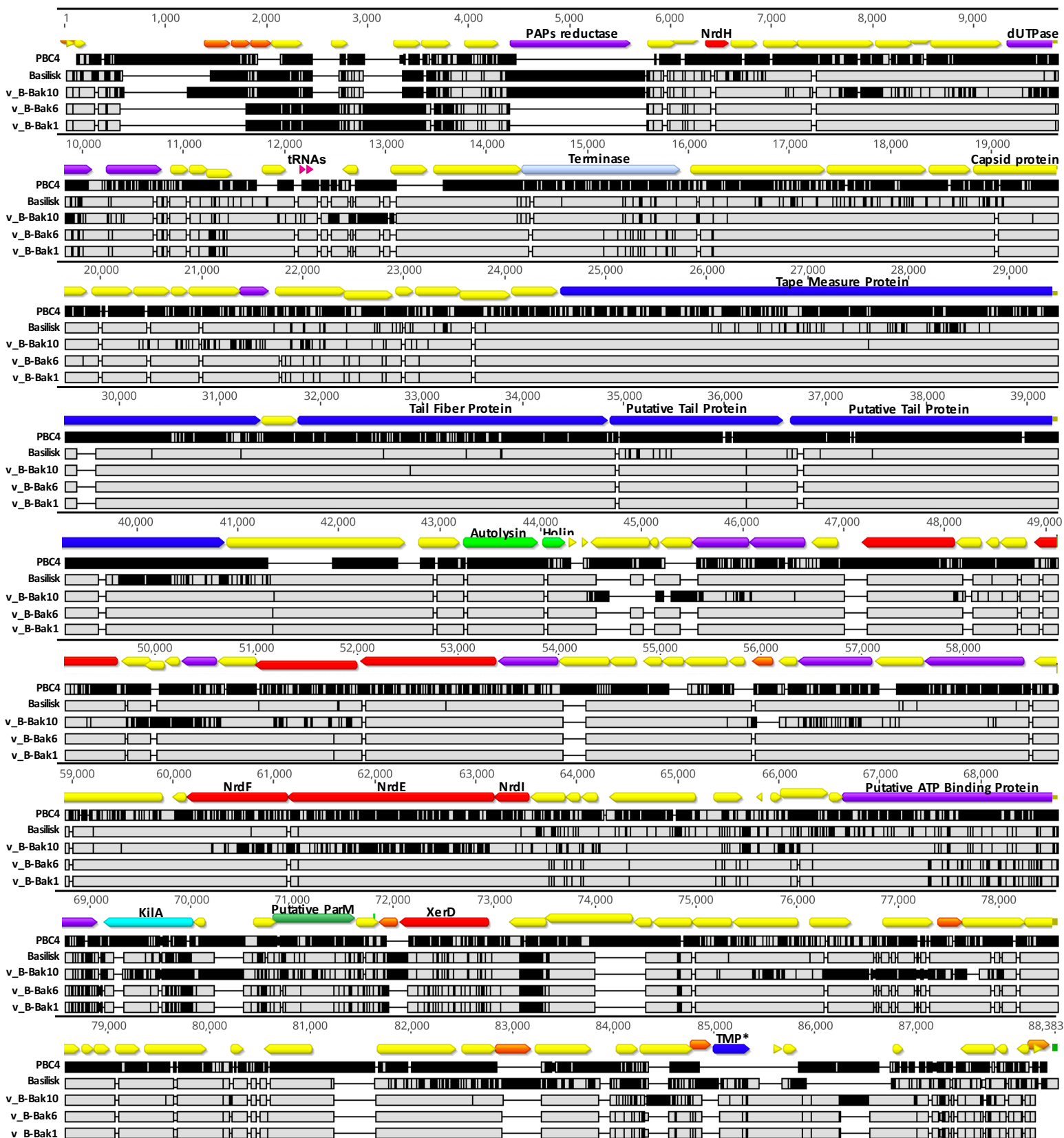

Supplement: Supplementary file 2 — Figure S2. Whole genome alignments of phage v_B-Bak1, v_B-Bak6, v_B-Bak10 and the Basilisk and PBC4 reference genomes. Nucleic acid sequence is designated by grey bars, SNP density is indicated by vertical black lines. Open Reading Frames (ORFs) are respective to the Basilisk phage genome are illustrated above the alignment for reference. (PDF 2006 kb) [file 12864_2018_5056_MOESM2_ESM.pdf]

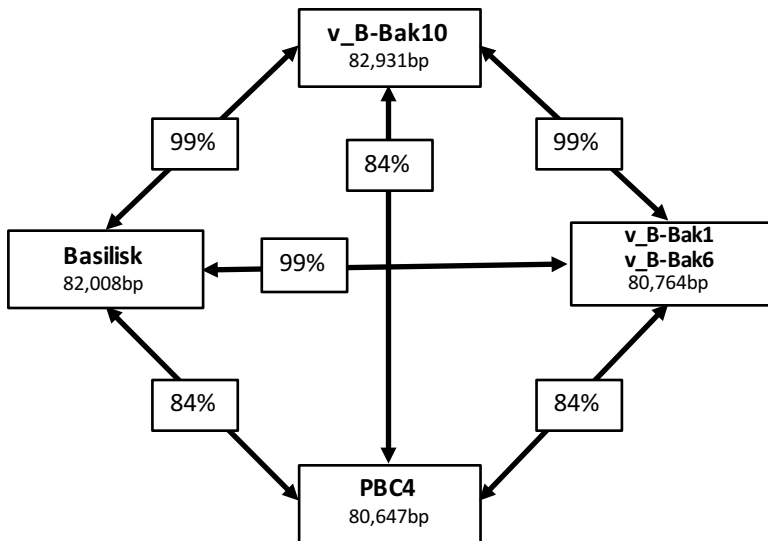

Supplement: Supplementary file 3 — Figure S3. Whole-genome percent similarity among the phage genomes analyzed. (PDF 11 kb) [file 12864_2018_5056_MOESM3_ESM.pdf]

A)

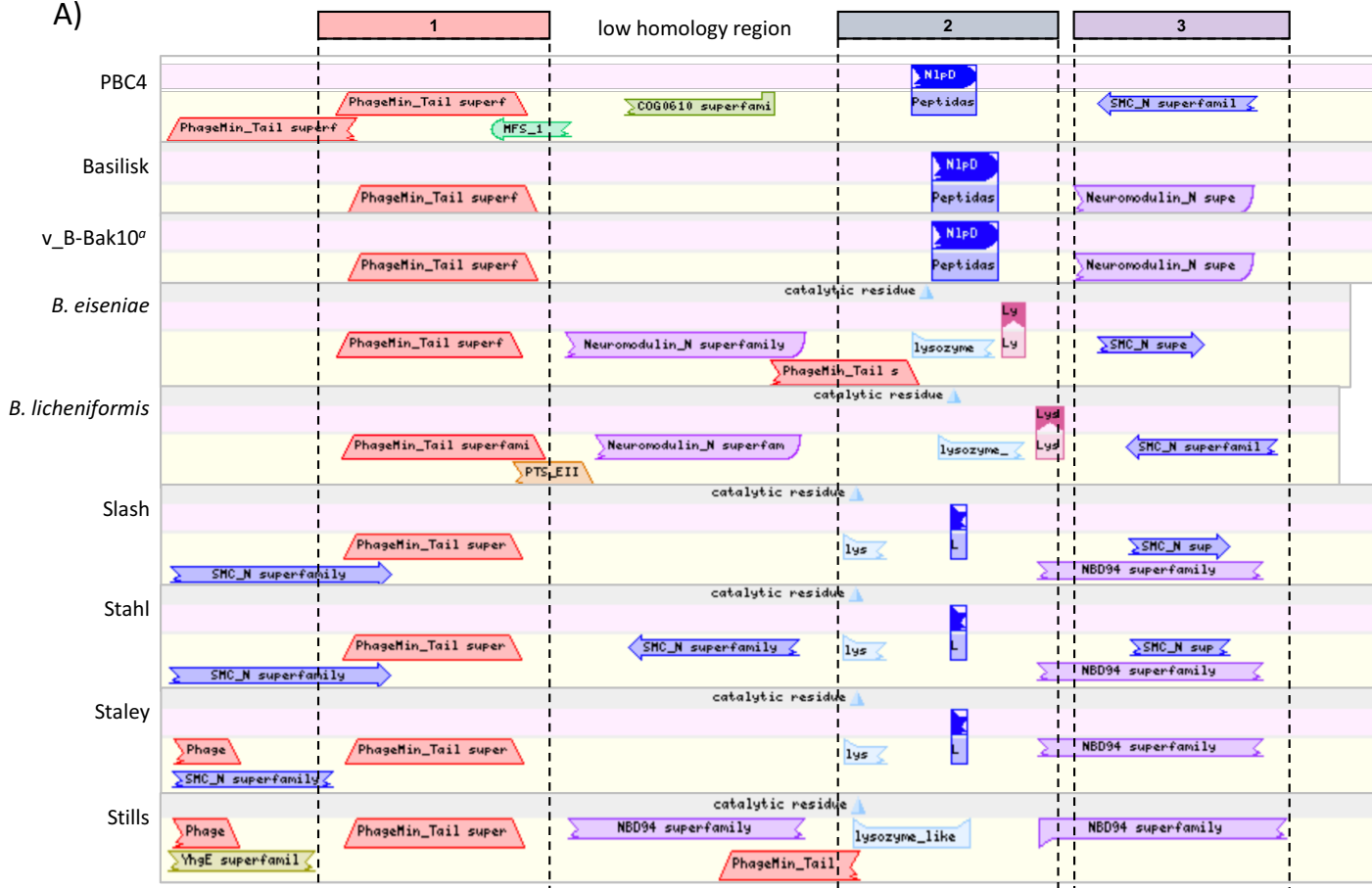

B)

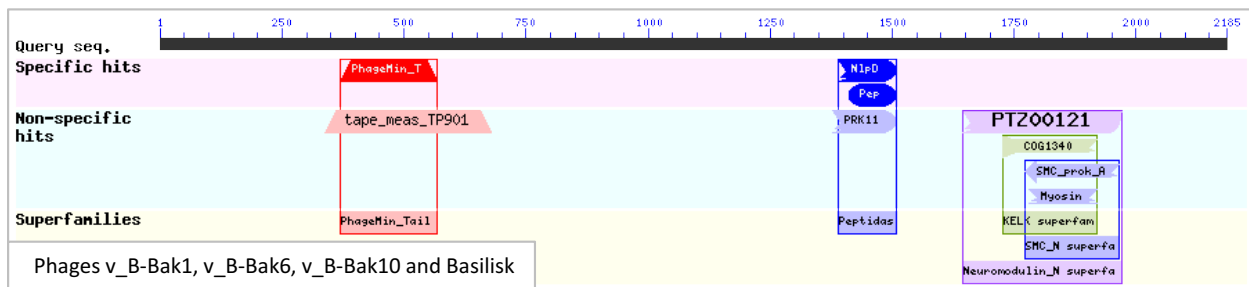

c)

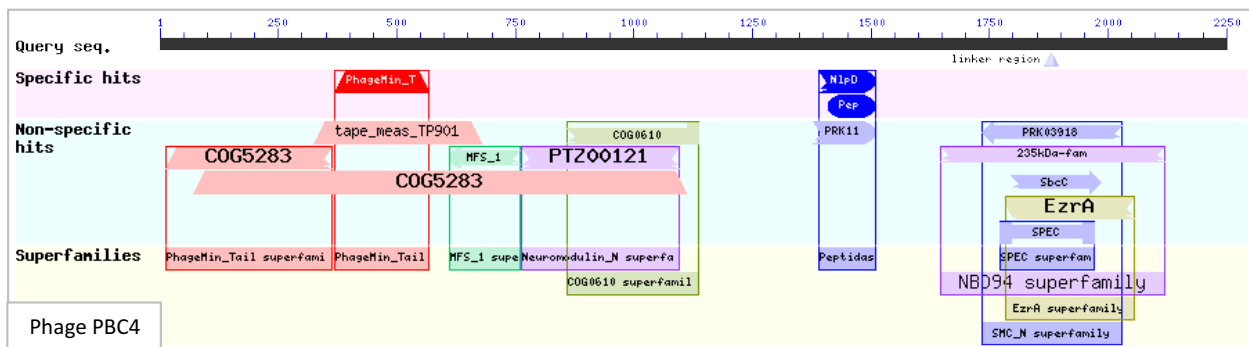

Supplement: Supplementary file 4 — Figure S4. Tape Measure Protein domain content and organization. (A) Physical locations of conserved family domains with the TMPs of the BLPs and near relatives. Boxes designate the three putative domain regions in the BLPs, including the N-terminal phage-related minor tail protein domain (box 1), peptidase domain (box 2), and C-terminal domains [3]. a phages v_B-Bak1, v_B-Bak6, v_B-Bak10 possess identical domain architectures, for brevity only v_B-Bak10 is shown here. Expanded views of all domain hits observed for v_B-Bak1, v_B-Bak6, v_B-Bak10, Basilisk are illustrated in (B) and phage PBC4 (C). (PDF 239 kb) [file 12864_2018_5056_MOESM4_ESM.pdf]

A)

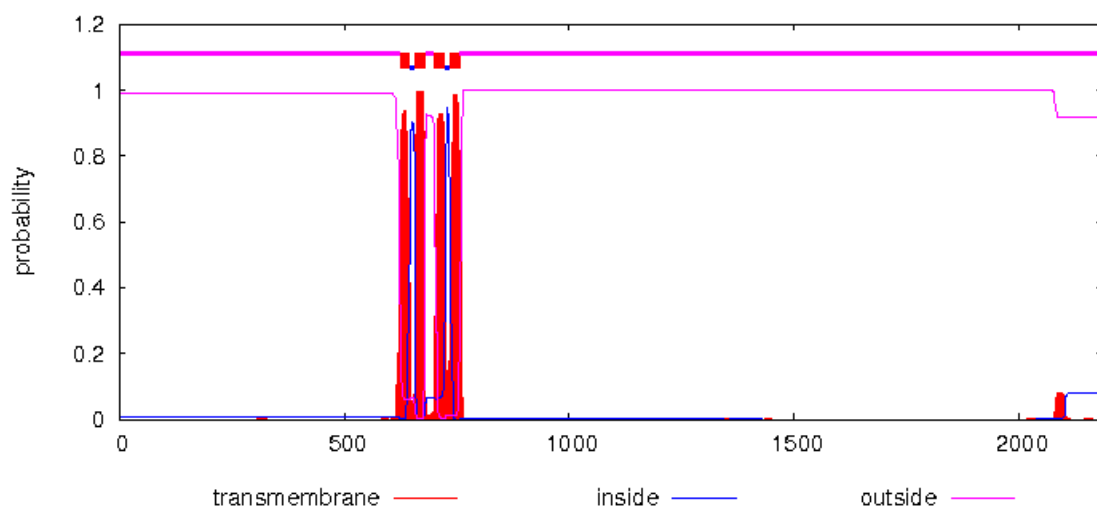

B)

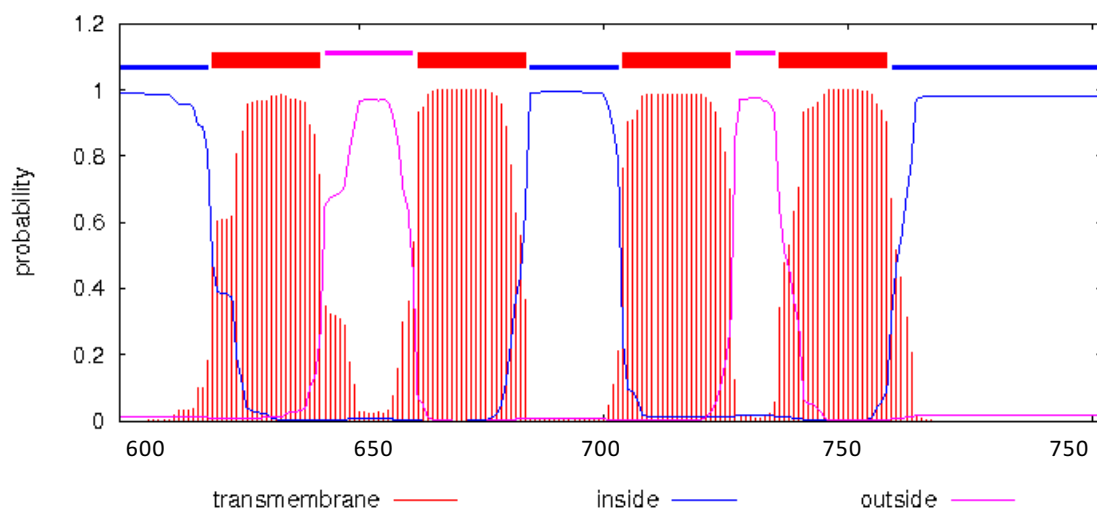

Supplement: Supplementary file 5 — Figure S9. Title of data: Predicted transmembrane helix domains (TMH) in the BLP TMPs Description of data: Predicted location and orientation of transmembrane helices in the TMP proteins of the BLPs, A) position of the predicted TMH domain, B) expanded view of (TMH) domain. (PDF 48 kb) [file 12864_2018_5056_MOESM5_ESM.pdf]

TMHMM posterior probabilities for test

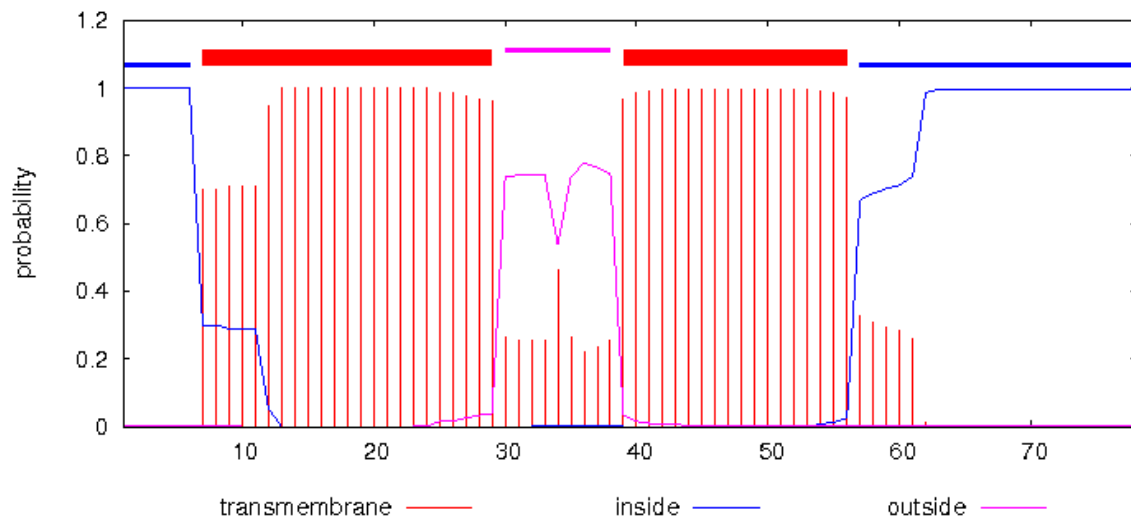

Supplement: Supplementary file 7 — Figure S10. Title of data: Predicated transmembrane helix domains (TMH) in BLP holin protein. Description of data: Predicted location and orientation of transmembrane helices in the holin proteins of the BLPs. (PDF 34 kb) [file 12864_2018_5056_MOESM7_ESM.pdf]

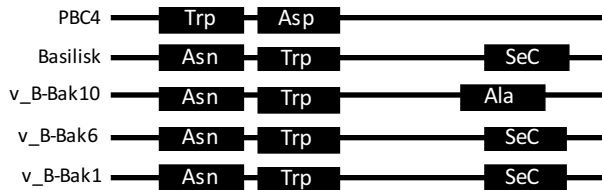

Supplement: Supplementary file 8 — Figure S5. Organization of putative tRNA genes encoded by the phages studied. (PDF 8 kb) [file 12864_2018_5056_MOESM8_ESM.pdf]

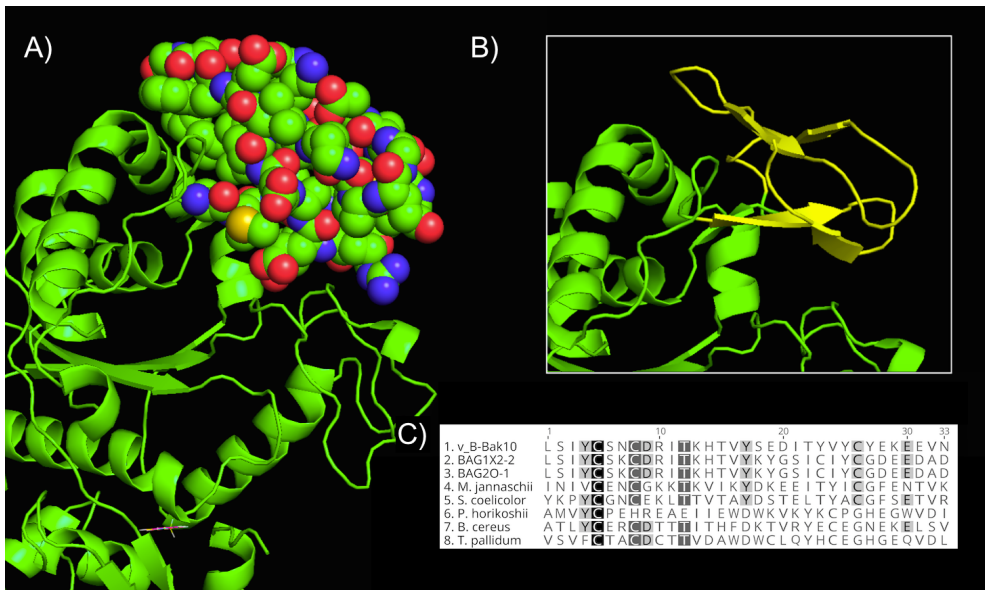

Supplement: Supplementary file 9 — Figure S6. Protein homology region of Aminoacyl-tRNA Synthetase (ARS) CP family domain encoded by phage v_B-Bak10. Crystal structure of the CP domain of bacterial ARS (A and B) and corresponding amino acid alignment of CP domain homologs (C). (PDF 722 kb) [file 12864_2018_5056_MOESM9_ESM.pdf]
