# Supplementary material for: Genomic characterization of three novel Basilisk-like phages infecting Bacillus anthracis
Source: BMC Genomics. 2018 Sep 18;19:685. doi: 10.1186/s12864-018-5056-4 (PMC6145125; doi:10.1186/s12864-018-5056-4)
Supplement: Supplementary file 6 — Figure S8. Conserved family domains within the BLP TFPs and endolysin proteins. The positions of the conserved family domains within the TFPs of Basilisk, _B-Bak1, v_B-Bak6, v_B-Bak10 phages (A) and PBC4 (B) are illustrated along with the conserved family domains within the endolysins of Basilisk, _B-Bak1, v_B-Bak6, v_B-Bak10 phages (D) and PBC4 (E). (PDF 620 kb) [file 12864_2018_5056_MOESM6_ESM.pdf]

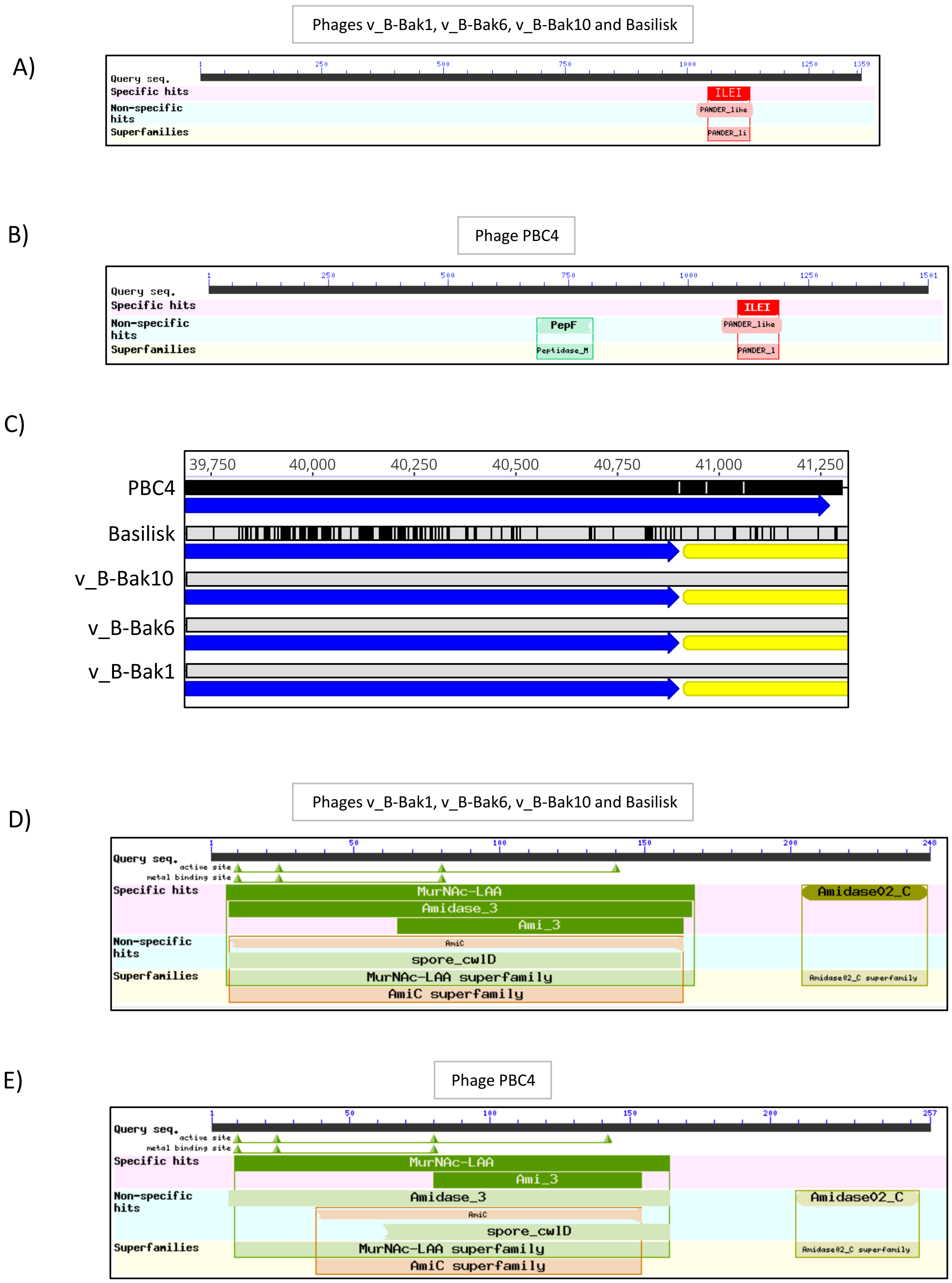
A)
Phages v_B-Bak1, v_B-Bak6, v_B-Bak10 and Basilisk
Query seq.
Specific hits
Non-specific hits
Superfamilies
ILEI
PANDER_like
PANDER_li
B)
Phage PBC4
Query seq.
Specific hits
Non-specific hits
Superfamilies
ILEI
PepF
PANDER_like
Peptidase_M
PANDER_l
C)
39,750
40,000
40,250
40,500
40,750
41,000
41,250
PBC4
Basilisk
v_B-Bak10
v_B-Bak6
v_B-Bak1
D)
Phages v_B-Bak1, v_B-Bak6, v_B-Bak10 and Basilisk
Query seq.
active site
metal binding site
Specific hits
MurNAc-LAA
Amidase_3
Ami_3
Amidase02_C
Non-specific hits
AmiC
spore_cwlD
Superfamilies
MurNAc-LAA superfamily
AmiC superfamily
Amidase02_C superfamily
E)
Phage PBC4
Query seq.
active site
metal binding site
Specific hits
MurNAc-LAA
Ami_3
Non-specific hits
Amidase_3
Amidase02_C
AmiC
spore_cwlD
Superfamilies
MurNAc-LAA superfamily
AmiC superfamily
Amidase02_C superfamily
