# Supplementary material for: Genomic characterization of three novel Basilisk-like phages infecting Bacillus anthracis
Source: BMC Genomics. 2018 Sep 18;19:685. doi: 10.1186/s12864-018-5056-4 (PMC6145125; doi:10.1186/s12864-018-5056-4)
Supplement: Supplementary file 10 — Figure S7. Amino acid sequence alignment of the putative DUTPase proteins encoded by the Basilisk-like phages. (PDF 3253 kb) [file 12864_2018_5056_MOESM10_ESM.pdf]

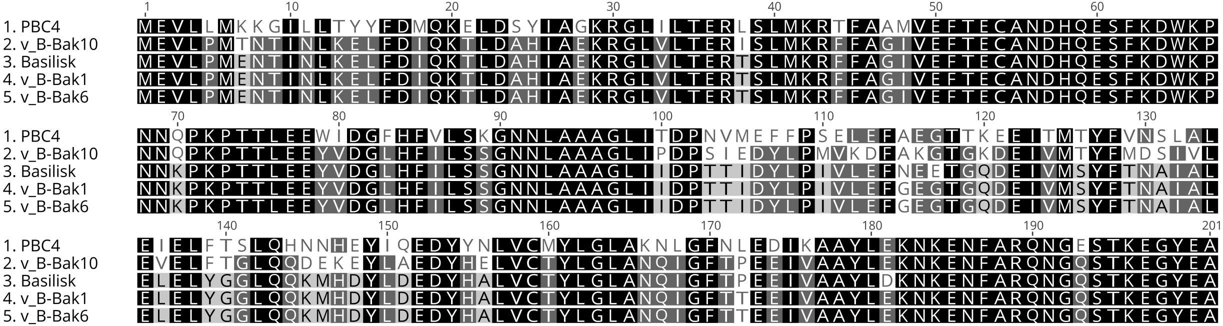
1 10 20 30 40 50 60
1. PBC4 MEVLLMKKGILLTYYFDMQKELDSYIAGKRGLILTERLSLMKRTFAAMVEFTECANDHQESFKDWKP
2. v_B-Bak10 MEVLPMTNTINLKELFDIQKTLDAHIAEKRGLVLTERISLMKRFFAGIVEFTECANDHQESFKDWKP
3. Basilisk MEVLPMENTINLKELFDIQKTLDAHIAEKRGLVLTERTSLMKRFFAGIVEFTECANDHQESFKDWKP
4. v_B-Bak1 MEVLPMENTINLKELFDIQKTLDAHIAEKRGLVLTERTSLMKRFFAGIVEFTECANDHQESFKDWKP
5. v_B-Bak6 MEVLPMENTINLKELFDIQKTLDAHIAEKRGLVLTERTSLMKRFFAGIVEFTECANDHQESFKDWKP
70 80 90 100 110 120 130
1. PBC4 NNQPKPTTLEEWIDGFHFVLSKGNNLAAAGLITDPNVMEFFPSELEFAEGTTKEEITMTYFVNSLAL
2. v_B-Bak10 NNQPKPTTLEEYVDGLHFILSSGNNLAAAGLIPDPSIEDYLPMVKDFAKGTGKDEIVMTYFMDSIVL
3. Basilisk NNKPKPTTLEEYVDGLHFILSSGNNLAAAGLIIDPTTIDYLPIVLEFNEETGQDEIVMSYFTNAIAL
4. v_B-Bak1 NNKPKPTTLEEYVDGLHFILSSGNNLAAAGLIIDPTTIDYLPIVLEFGEGTGQDEIVMSYFTNAIAL
5. v_B-Bak6 NNKPKPTTLEEYVDGLHFILSSGNNLAAAGLIIDPTTIDYLPIVLEFGEGTGQDEIVMSYFTNAIAL
140 150 160 170 180 190 201
1. PBC4 EIELFTSLQHNNHEYIQEDYYNLVCMYLGLAKNLGFNLEDIKAAYLEKNKENFARQNGESTKEGYEA
2. v_B-Bak10 EVELFTGLQQDEKEYLAEDYHELVCTYLGLANQIGFTPEEIVAAYLEKNKENFARQNGQSTKEGYEA
3. Basilisk ELELYGGLQQKMHDYLDEDYHALVCTYLGLANQIGFTPEEIVAAYLDKNKENFARQNGQSTKEGYEA
4. v_B-Bak1 ELELYGGLQQKMHDYLDEDYHALVCTYLGLANQIGFTTEEIVAAYLEKNKENFARQNGQSTKEGYEA
5. v_B-Bak6 ELELYGGLQQKMHDYLDEDYHALVCTYLGLANQIGFTTEEIVAAYLEKNKENFARQNGQSTKEGYEA
